# Efficacy of a Smartphone App Intervention for Reducing Caregiver Stress: Randomized Controlled Trial

**DOI:** 10.2196/17541

**Published:** 2020-07-24

**Authors:** Matthew Fuller-Tyszkiewicz, Ben Richardson, Keriann Little, Samantha Teague, Linda Hartley-Clark, Tanja Capic, Sarah Khor, Robert A Cummins, Craig A Olsson, Delyse Hutchinson

**Affiliations:** 1 Deakin University Geelong Australia; 2 Nous Group Melbourne Australia; 3 Policy & Planning, Barwon Child Youth & Family Geelong Australia; 4 Neurodevelopment and Disability Royal Children’s Hospital Melbourne Australia; 5 Murdoch Children’s Research Institute Centre for Adolescent Health Melbourne Australia; 6 Department of Paediatrics, University of Melbourne Melbourne Australia; 7 National Drug and Alcohol Research Centre University of New South Wales Sydney Australia

**Keywords:** mHealth, mobile phone, caregiver, psychological stress, mental health

## Abstract

**Background:**

Caregivers play a pivotal role in maintaining an economically viable health care system, yet they are characterized by low levels of psychological well-being and consistently report unmet needs for psychological support. Mobile app–based (mobile health [mHealth]) interventions present a novel approach to both reducing stress and improving well-being.

**Objective:**

This study aims to evaluate the effectiveness of a self-guided mobile app–based psychological intervention for people providing care to family or friends with a physical or mental disability.

**Methods:**

In a randomized, single-blind, controlled trial, 183 caregivers recruited through the web were randomly allocated to either an intervention (n=73) or active control (n=110) condition. The intervention app contained treatment modules combining daily self-monitoring with third-wave (mindfulness-based) cognitive-behavioral therapies, whereas the active control app contained only self-monitoring features. Both programs were completed over a 5-week period. It was hypothesized that intervention app exposure would be associated with decreases in depression, anxiety, and stress, and increases in well-being, self-esteem, optimism, primary and secondary control, and social support. Outcomes were assessed at baseline, postintervention, and 3-4 months postintervention. App quality was also assessed.

**Results:**

In total, 25% (18/73) of the intervention participants were lost to follow-up at 3 months, and 30.9% (34/110) of the participants from the wait-list control group dropped out before the postintervention survey. The intervention group experienced reductions in stress (*b*=−2.07; *P*=.04) and depressive symptoms (*b*=−1.36; *P*=.05) from baseline to postintervention. These changes were further enhanced from postintervention to follow-up, with the intervention group continuing to report lower levels of depression (*b*=−1.82; *P*=.03) and higher levels of emotional well-being (*b*=6.13; *P*<.001), optimism (*b*=0.78; *P*=.007), self-esteem (*b*=−0.84; *P*=.005), support from family (*b*=2.15; *P*=.001), support from significant others (*b*=2.66; *P*<.001), and subjective well-being (*b*=4.82; *P*<.001). On average, participants completed 2.5 (SD 1.05) out of 5 treatment modules. The overall quality of the app was also rated highly, with a mean score of 3.94 out of a maximum score of 5 (SD 0.58).

**Conclusions:**

This study demonstrates that mHealth psychological interventions are an effective treatment option for caregivers experiencing high levels of stress. Recommendations for improving mHealth interventions for caregivers include offering flexibility and customization in the treatment design.

**Trial Registration:**

Australian New Zealand Clinical Trial Registry ACTRN12616000996460; https://www.anzctr.org.au/Trial/Registration/TrialReview.aspx?id=371170

## Introduction

### Background

Caring for people living with physical or mental health difficulties can be a challenging role, one that is becoming increasingly common as trends in public policy move toward assisting people with disabilities to remain within their family environment for as long as possible [1]. A caregiver, or informal carer, is defined as a person who provides any informal, ongoing assistance to people with disabilities, including physical conditions and mental and behavioral disorders, such as developmental disability, or to older people (aged ≥65 years) [2]. Caregivers provide substantial social and economic contributions to their community, with approximately 2.7 million caregivers in Australia (12% of the population), 43.5 million in the United States (18% of the population), and 6.5 million in the United Kingdom (8% of the population), contributing over US $60 billion in unpaid care and support per year [2-5]. In Australia, over 50% of the caregivers provide care for more than 20 hours per week, which affects their capacity to participate in the workforce [2]. As a result, carers have a median weekly income estimated to be 42% lower than noncarers and experience limitations in opportunities for social connection and support [1,2].

Despite their challenging circumstances, caregivers have been reported to identify positive aspects associated with caregiving, including a sense of value in their role [6,7]. However, there can be costs to a caregiver’s subjective and objective well-being, particularly when the burden of care is high. The rates of mental and physical ill-health are substantially higher in caregivers than noncaregivers [1,8,9], including elevated symptoms of stress, depression, and anxiety; higher rates of psychiatric disorders; and reduced overall subjective well-being [10-12]. Notably, high rates of depression, anxiety, and stress have been reported in caregivers supporting people with intellectual disability [1], dementia [12,13], Parkinson disease [14], chronic childhood illness [15], autism [16], and a psychiatric disorder [17], alongside other forms of disability [18]. Caregiver stress is a particular concern when care recipients are affected by long-term or terminal illnesses, major cognitive impairment, or additional behavioral and emotional problems beyond the core symptomatology of their condition [1,19].

Compromised emotional well-being in caregivers (eg, mental disorders) may adversely impact care recipients. There is evidence, for example, that care recipients have poorer general health, mental health, and quality of life and exacerbated disability symptomatology when caregivers experience mental health problems [20,21]. The caregiver burden has also been associated with poorer caregiving quality, including the use of coercive or harmful management techniques, which may damage the relational bond between a caregiver and the care recipient [22-24]. Such relationships are likely to be bidirectional: more complex caregiving contexts may increase caregiver burden, and vice versa [20]. Furthermore, the experience of caregiver psychological difficulties is a risk factor for a breakdown in care and a shift to formal care arrangements, such as placement in a supervised care environment [25,26]. Thus, there is a growing recognition of the need to adopt a family systems approach to support people with disabilities and their caregivers alike [1].

Given the available evidence on the significance of caregiver burden, tailored interventions designed to reduce stress and promote well-being in carers are critically important. Among existing interventions, the primary psychological treatments are based on principles of cognitive behavioral therapy (CBT), which have been shown to reduce depression in caregivers [27,28]. Although the effects on anxiety and stress have received less attention, the extant literature is equivocal [12]. Previous studies have found that cognitive reframing may be particularly effective for reducing subjective stress, anxiety, and depressive symptomatology in caregivers [29,30]. This technique may be most pertinent in challenging unrealistic, self-defeating, and distressing cognitions about either the caregiving role or the care recipients’ behavior or condition. Another promising CBT technique for caregiver mental health is behavioral activation, whereby an individual is assisted to engage in enjoyable and meaningful activities and thereby develop or reconnect with gratifying or valued aspects of their lives [31,32]. Behavioral activation may help address the activity restriction commonly experienced by caregivers, a known depression risk [33]. Third-wave CBT techniques focusing on thoughts and emotions, such as mindfulness-based interventions, acceptance and commitment therapy, and dialectical behavior therapy, have also demonstrated efficacy in reducing a range of mental health conditions [34], including stress in caregivers of people with dementia [35], intellectual or developmental disabilities [36], and palliative illness [37]. Such approaches may be particularly useful for caregivers, encouraging acceptance of negative thoughts and emotions without judgment.

Although these approaches show promise, caregivers face a number of barriers to accessing in-person treatment programs, including economic, geographic, and mobility factors; limited time to engage in interventions; and difficulties in finding and/or affording the cost of suitable alternative caregiver support to attend treatment [38-40]. Furthermore, caregivers often report difficulties in prioritizing their own needs or setting aside time for *nonessential* activities, which may include treatment interventions [41]. Digital technologies may help address issues of accessibility to treatment, particularly when there are barriers to attending the more traditional face-to-face individual or group interventions. The benefits of digital programs include reduced costs, increased availability (particularly in geographical locations where services may be restricted), as well as convenience of use compared with traditional formats [42-44]. However, research is needed to determine the extent to which evidence-based techniques can be adapted to these new media platforms while preserving their efficacy in caregiver populations. A number of interventions have successfully adapted CBT techniques to digital platforms for carers using video teleconferencing, websites with text and/or web-based video education and coaching, and online discussion group technologies. Mobile app–based interventions are notably absent from the caregiver intervention literature, with the research needed to examine whether brief interventions, delivered through a mobile phone, can realistically deliver a usable service to caregivers.

Mobile app–based brief interventions offer a number of strengths over other digital delivery platforms. Their small size and portability allow an intervention to be readily accessed at times of greatest need [45]. Their use also allows for real-time symptom and activity monitoring, together with assessment of treatment progress via ecological momentary assessment (EMA) as well as the provision of personalized feedback [46]. Emerging literature suggests that psychological interventions delivered via smartphone devices can reduce anxiety [47], depression [48], and stress [49-51] and improve well-being [52] in the general population. To our knowledge, such interventions have not yet been trialed with caregivers.

### Aims and Hypotheses

This study is a randomized controlled trial of a mobile app–based, self-directed psychological intervention for people who are providing care to family or friends with a physical and/or mental disability. It was hypothesized that the intervention would produce a greater reduction in stress, depression, and anxiety as well as increased well-being, compared with control participants (hypothesis 1). To assess the broader impact, we also evaluated emotional well-being, self-esteem, optimism, primary and secondary control, and perceived social support (secondary outcomes; hypothesis 2). We hypothesized that these improvements in self-reports will be maintained for 3 months postintervention for primary outcomes (hypothesis 3) and secondary outcomes (hypotheses 4). Although the intervention was designed to provide a range of modules with different techniques that could each be useful for improving outcomes, we tested the possibility that the effect of intervention allocation was moderated by the number of treatment modules completed. In particular, we predicted that improvements in primary and secondary outcomes would be stronger for individuals allocated to the treatment condition who engage in more modules (hypothesis 5). We also explored the usefulness of this form of intervention through caregivers’ perceptions of the app’s engagement, functionality, aesthetics, information, and quality, expecting positive ratings across these metrics for the intervention (hypothesis 6).

## Methods

### Design

The design of the trial was a 2 (condition: *StressLess* intervention and *StressMonitor* active control) × 3 (occasion: baseline, postintervention, and 4-month follow-up), parallel, single-blind, randomized controlled trial. This study was approved by the Deakin University Human Research Ethics Committee (2016-151) and registered with the Australian New Zealand Clinical Trials Registry (ACTRN12616000996460). A Consolidated Standards of Reporting Trials (CONSORT) checklist for this study is available in Multimedia Appendix 1. Furthermore, the CONSORT eHealth document [53] is also included in Multimedia Appendix 2.

### Participants

Participants were recruited through a mix of traditional strategies and targeted social media advertising. Support was sought from caregiver organizations and services, who agreed to display study flyers (both in physical and digital forms) and allowed the research team to attend caregiver events and seminars for recruitment purposes. Social media advertising was conducted through Facebook, with separate advertisement campaigns targeting either Australians broadly or those with an interest in specific disability topics (eg, *Attention deficit hyperactivity disorder awareness*, *Alzheimer’s awareness*, and *physical disability*). Campaigns were restricted to adult Facebook users located in Australia accessing the platform through an Apple iOS device. Although the advertisements did not immediately identify the institutional affiliations of the research team, this was made clear in the plain language statement that participants were directed to via weblinks to start the program.

**Figure 1:**
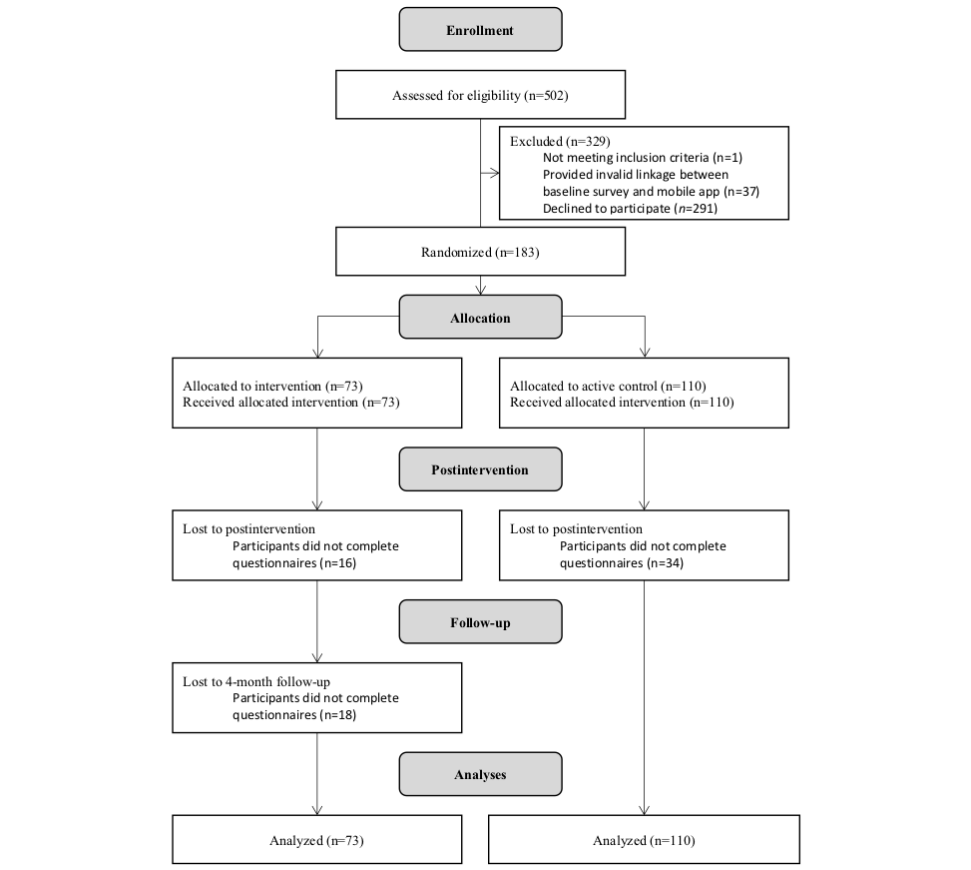
This Figure provides a CONSORT flow chart of participant numbers.

To be eligible to join the study, participants were required to be (1) an Australian resident, (2) aged 18 years or older, (3) fluent in English, (4) helping to support a friend or relative with a physical or mental condition/disability, (5) able to access an Apple iOS mobile phone device (iPhone or iPad) with internet access for the duration of the study, and (6) not have participated in an electronic health (eHealth) intervention (any technology-based health intervention, including mobile apps) within the previous 6 months. Smartphone app literacy was also a de facto eligibility criterion but was assumed by the participant’s willingness to sign up for the study. A CONSORT flow diagram is provided below (Figure 1). Recruitment to the baseline component of the study ran from September 2016 to April 2017.

### Sample Size Calculation

The required sample size was powered with the following assumptions: (1) a moderate group difference (SD 0.5) between the intervention and active control groups for the primary and secondary outcomes at postintervention; (2) power set at 0.80; (3) α set at .05 (2 tailed); (4) expected attrition rate of 20% for the intervention group [54]; and (5) an allocation ratio of 3:2 (active control: intervention) under the expectation that attrition would be around 30% for the active control group, as they only receive self-monitoring features of the app and not intervention content during the control phase. Under these assumptions, the adjusted target sample size at baseline was 68 and 100 for the intervention and active control groups, respectively.

### Intervention: StressLess

**Figure 2:**
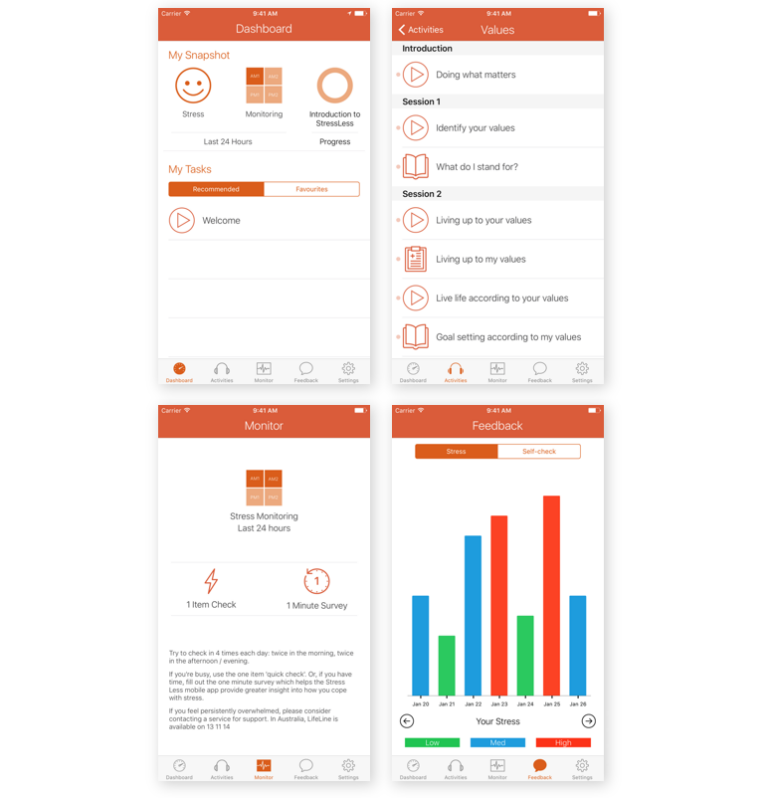
This figure shows layout of the app.

StressLess is a 5-week, self-directed intervention, based on the principles of second- and third-wave CBTs [55], delivered through a mobile app (Figure 2). The intervention provides psychoeducation (through text, video, audio, and graphics) and a series of interactive exercises or activities. The intervention comprises 5 modules (detailed in Multimedia Appendix 1): (1) an introduction involving psychoeducation about stress reduction and third-wave CBT; (2) values clarification and goal setting; (3) mindfulness skills involving observation of the self and connection with the present moment, cognitive diffusion, and acceptance; (4) well-being enhancement through positive psychology techniques and cognitive restructuring; and (5) behavioral activation to increase engagement in, and enjoyment of, pleasant or valued activities. A *Troubleshooting* tab was also available beyond the core intervention modules, which contained a series of activities to help with stress (eg, destress with a body scan and breathing to diffuse negative thoughts). The intervention content was designed to provide a suite of therapeutic techniques with demonstrated efficacy in the broader literature, enabling participants’ autonomy in selecting the techniques that they feel work best for them. Participants could work through the modules at their own pace and in any order across the 5 weeks, but they were encouraged to complete one module per week in a recommended sequence. Each module that a participant completed was logged by the app to enable tracking of how many modules a participant tried.

In addition to the intervention modules, StressLess also supports users in self-monitoring their well-being through in-the-moment assessments (EMA). Participants were prompted to complete a self-monitoring assessment up to 4 times per day via the StressLess app notification function. Participants were prompted to complete either a *1 item check* asking them to rate their current stress levels or a longer *1 min check* assessing whether the participant had experienced a stressful event in the previous 30 min. The *1 min check* involved assessments of coping and positive and negative affect. Coping was assessed using an eight-item checklist of various coping strategies (eg, *distracted myself*) for the recent stressful event. Momentary positive and negative effects were assessed using the Homeostatically Protected Mood Scale, described in *Measures* section [56,57]. Users were able to adjust the notification settings of the self-monitoring component, with the default number of sampling points set at 2 times per day (one in the morning after 8 AM and one in the afternoon/night before 10 PM). Self-monitoring EMA data were automatically collated by the mobile app and presented to users through a feedback bar chart. Source code for StressLess is available on request from the corresponding author.

### Active Control: StressMonitor

The active control involved the mobile app StressMonitor. This comprised the same self-monitoring EMA function and feedback bar chart as the StressLess intervention but did not contain any intervention modules. The inclusion of an active control condition enabled the statistical separation of effects because of novelty (or burden) of completing app-based self-monitoring of mood from treatment outcomes.

### Measures

#### Demographics

Items assessing participants’ caregiver roles were adapted from the Australian Bureau of Statistics’ (ABS) Survey of Disability, Aging, and Carers (SDAC) [58]. These assessed the impact of a participant’s caregiver role in terms of the respondent’s time, energy, emotions, finances, and daily activities, with response options ranging from *1* (*not at all*) to *4* (*a lot*)*.* The care recipients’ disability type was also assessed using the ABS’ SDAC categories of sensory (eg, loss of sight and loss of hearing), intellectual (difficulty learning or understanding things), physical (eg, shortness of breath and chronic or recurrent pain or discomfort), psychosocial (eg, social or behavioral difficulties, memory problems, or periods of confusion), head injury/stroke, acquired brain injury, or other long-term conditions [58]. Importantly, all disability conditions were required to be long term and restrict the care recipients’ everyday activities.

#### Primary Intervention Outcomes

The primary outcomes were the participants’ stress levels, depression, anxiety, and subjective well-being. The first 3 variables were measured using the Depression Anxiety Stress Scale-21 [59]. This scale contains 21 items, separated into 3 subscales, assessing the self-reported frequency/severity of emotional states over the past week. The *depression* subscale contains items assessing the symptomatology of mood disorders, including hopelessness, low self-esteem, and low positive affect, for example, “I felt downhearted and blue” [59,60]. The *anxiety* subscale assesses the symptomatology of panic disorders through items on autonomic arousal, physiological hyperarousal, and the subjective feelings of fear, for example, “I was aware of dryness in my mouth.” The *stress* subscale assesses tension, agitation, and negative affect, for example, “I found it hard to wind down.” Higher subscale scores indicate more frequent/severe emotional states. The Depression Anxiety Stress Scale-21 has demonstrated robust psychometric properties, with the three-scale solution and internal consistency (α>.78) being supported in Australian samples in the pen-and-paper form [60] and via web-based survey [61]. In this study, subscale-level internal consistency estimates ranged from 0.67 to 0.83 for anxiety, from 0.75 to 0.88 for depression, and from 0.67 to 0.81 for stress (full results provided as Multimedia Appendix 2).

The Personal Wellbeing Index (PWI) was used to assess the primary intervention outcome of participants’ subjective well-being [62]. PWI consists of 7 items asking respondents to rate how satisfied they are across 7 life domains: standard of living, personal health, achieving in life, personal relationships, personal safety, community connectedness, and future security. The ratings are made across an 11-point scale, ranging from *0* (*no satisfaction at all*) to *10* (*completely satisfied*), with higher scores indicating higher satisfaction*.* In addition, a total score was calculated from the 7 PWI items, scaled from 0 to 100. Psychometric evaluations of the PWI have demonstrated acceptable reliability (α>.77) and factorial validity in Australian populations [63] as well as internationally [62]. Acceptable factor structure and internal consistency have also been achieved via web-based collection of PWI data [64]. In this study, internal consistency ranged from 0.71 to 0.90 across groups and time.

#### Secondary Outcomes

Beyond the primary outcome measures listed earlier, the study also assessed additional secondary variables that were predicted to improve after completing the intervention. Affective mood was assessed using the Homeostatically Protected Mood Scale [56,57]. Respondents were asked to rate how well 3 positive affective terms (content, happy, and alert) describe their feelings about their life in general, rated using an 11-point scale, ranging from *0* (*not at all*) to *10* (*extremely*), with higher scores on each indicating that higher affective mood psychometric evaluations of the Homeostatically Protected Mood Scale with Australian samples (both via pen-and-paper survey [57] and through the web [65]) have demonstrated strong internal consistency (α=.85) [57,65] and convergent validity with other well-being measures, such as the PWI (*r*s=0.58-0.72) [57] and Satisfaction with Life scale (*r*=0.79) [65]. In this study, internal consistency ranged from 0.75 to 0.87 across groups and time.

Self-esteem was assessed using the Rosenberg Self-Esteem Scale [66]. This scale consists of 10 items assessing self-esteem (eg, “At times I think I am no good at all”), with response options completed using a 4-point scale, ranging from *1* (*strongly disagree*) to *4* (*strongly agree*)*.* In an Australian sample, the measure has demonstrated excellent test-retest reliability (*r*s=0.53-0.69 over 4 years) and internal consistency (α>.85) and was shown to correlate with constructs theoretically related to self-esteem, such as self-compassion (*r*s=0.36-0.63) [67]. For this study, Rosenberg’s original 5 positive items were included, thereby a single construct best described as positive self-esteem, with higher scores indicating higher self-esteem [66]. In this study, internal consistency ranged from 0.65 to 0.86 across groups and time.

Optimism was assessed using the *optimism* subscale from the Life Orientation Test-Revised [68]. This subscale comprises 3 items that measure the respondents’ generalized expectation of good outcomes in life, for example, “In uncertain times, I expect the best.” The responses are provided using a five-point scale, ranging from *0* (*strongly disagree*) to *4* (*strongly agree*), with higher scores indicating higher optimism*.* The measure has demonstrated acceptable psychometric properties in an Australian context with data collected through the web, including internal consistency (α>.80) [68,69], and convergent validity with measures of life satisfaction (*r*=0.4) [68] and quality of life (*r*=0.5) [70]. In this study, internal consistency ranged from 0.62 to 0.84 across groups and time.

Primary and secondary control were assessed using an abbreviated version of the Primary and Secondary Control Scale (PSCS) [71,72]. The PSCS consists of 25 items assessing specific cognitive and behavioral strategies aimed at either control of environmental circumstances (primary control; eg, “when bad things happen, I put lots of time into overcoming it”) or control of internal states (secondary control; eg, “when bad things happen, I ignore it by thinking about other things”), to minimize psychological impacts. The response options were completed using an 11-point scale, ranging from *0* (*do not agree at all)* to *10* (*agree completely*), with higher scores indicating higher primary/secondary control. This study adapted the measure by selecting a subset of the items as the two-factor solution developed by Cousins with an Australian sample [71]. This comprised 5 items for the secondary control subscale from Cousins’ [71] avoidant control subscale, and the 6 best performing items were selected to form the primary control subscale from Cousins’ approach control subscale. Cousins [71] demonstrated acceptable internal consistency (α>.72) for these 2 subscales in an Australian sample. In this study, internal consistency ranged from 0.75 to 0.87 for primary control and from 0.61 to 0.76 for secondary control across groups and time.

Social support was assessed using the Multidimensional Scale of Perceived Social Support [73]. This comprises 12 items assessing the perceived adequacy of support from family, friends, and significant other (eg, “I have a special person who is a real source of comfort to me”). Responses are recorded on a 7-point scale, ranging from *1* (*very strongly disagree*) to *7* (*very strongly agree*), and scoring is calculated for 3 subscales reflecting the 3 social support sources of (1) *family*, (2) *friends*, and (3) *significant* *other*. Higher scores indicate higher perceived social support from each social support source. Within an Australian sample, the Multidimensional Scale of Perceived Social Support has demonstrated strong internal consistency (α=.90) and stability over a 1-year testing period (*r*=0.61) [74]. In this study, subscale-level internal consistency estimates ranged from 0.75 to 0.93 for family support, from 0.80 to 0.92 for support from friends, and from 0.80 to 0.93 for social support from others.

#### App Quality

The quality of the intervention app was assessed using the Mobile Application Rating Scale [75]. This scale comprises 23 items rated on a 5-point rating scale. The Mobile Application Rating Scale consists of 4 subscales: engagement, functionality, aesthetics, and information. The mean item score across the 4 subscales was used to determine an *objective measure of the overall quality* of the app, with higher scores indicating higher app quality. Furthermore, the Mobile Application Rating Scale also includes a subscale assessing the *subjective quality* of the app, consisting of items assessing whether the participant would recommend the app to others, plans to use the app again in the next 12 months, would pay to use the app, and their overall rating of the app out of 5. In this study, an adapted version of the Mobile Application Rating Scale was used, excluding the items assessing the entertainment value and evidence base for the app. These items were removed from the mean score calculation according to the guidelines [76].

### Procedure

After providing informed consent via Qualtrics (by reading a plain language statement and then responding to a question about whether they consented) and meeting the study eligibility criteria, participants were invited to complete the baseline assessment as a web survey. Participants were then randomly allocated to either the active control or intervention arm using a 3:2 assignment in blocks of 5 created through Qualtrics (web-based survey provider of choice), with the expectation that attrition would be higher in the active control group because of lower incentive to remain in the study. Instructions were provided to participants detailing how to install the app (either StressLess or StressMonitor) on their mobile phone or iPad. The app is free and does not include any hidden costs. For both groups, the plain language statement provided via the baseline Qualtrics survey provided contact details for free helplines if the participants felt distressed at any stage because of the intervention. The StressLess and StressMonitor apps also contained these contact details in the app to remind participants that they could contact LifeLine (a free, Australian counseling service) if they felt distressed.

Following the download of the app, participants then completed 5 weeks with their assigned app, with weekly contact from the research team by either an email or phone call to maximize engagement. In more detail, a standard email was sent to all participants in each group in weeks 1, 2, 4, and 5 explaining an aspect of either the intervention or the active control program. For example, week 2 emails were titled *Mindfulness with StressLess* and *Mood Monitoring: How does your mood change across the day?* for the intervention and active control conditions, respectively. In addition, participants were contacted through a phone call in week 3 to answer any queries about the use of the app. These phone calls were used to identify any technical difficulties and to maintain engagement. They were not designed for therapeutic purposes.

Participants then completed the postintervention assessment as a web survey and were reimbursed for their time with a $50 voucher. The postintervention survey was identical for participants from both groups, with the exception that the intervention group received the app quality measure. Furthermore, active control participants who completed the postintervention survey were provided with instructions on how to download the intervention app from the iOS app store. Finally, intervention participants were invited to complete a follow-up survey 4 months after completing the postintervention assessment.

### Analysis

Following the principles of intention-to-treat (ITT) analysis, individuals were retained in the group they were randomized to. Thus, even in cases where participants in the intervention group did not use the app at all (n=15), they were retained in the intervention group for the purposes of analysis. Missing data were handled using multiple imputation, with 50 imputations. By default, Mplus uses Monte Carlo Markov Chains with 100 iterations per imputation and chained equations to impute missing values for variables [77]. These imputed files were then imported into Mplus version 8 for multilevel modeling to test (1) the efficacy of the intervention compared with the control condition across study variables at postintervention for primary outcomes (hypothesis 1) and secondary outcomes (hypothesis 2), (2) the maintenance of treatment effects at the 4-month follow-up assessment for primary outcomes (hypotheses 3) and secondary outcomes (hypotheses 4), and (3) the impact of the number of modules completed on treatment efficacy (dose-response effects; hypothesis 5).

For the evaluation of efficacy, time was entered as a level 1 predictor (0=baseline and 1=postintervention). At level 2, group (0=control and 1=intervention) was included as a predictor of the dependent variable (DV) as well as a moderator of the level 1 relationship between time and DV scores. This latter effect (a cross-level interaction) was used to ascertain whether the rate of improvement in symptoms was greater for intervention participants than for those in the control group (hypotheses 1 and 2). Maintenance effects were tested similarly, although the time effect compared postintervention (coded 0) against the 4-month follow-up time point (coded 1; hypotheses 3 and 4). As the follow-up data were only collected for the intervention group, there was no level 2 predictor for group. Dose-response effects were tested with the intervention group only, by moderating the time effect by the number of modules completed (hypothesis 5). Each outcome variable was modeled separately. Descriptive statistics were reported for the evaluation of user ratings of the intervention (hypothesis 6). All effects were tested at *P*=.05 (two-tailed) unless otherwise indicated.

## Results

### Sample Characteristics and Caregiving Context

The final sample consisted of 183 caregivers; Table 1 shows the demographic characteristics of the sample. The average participant was female (174/183, 95.1%), aged 39.5 (SD 6.27) years, and provided full-time home care to a child with a disability (145/183, 79.2%). The majority (107/183, 58.4%) of the participants reported that their care recipient received government funding for disability support, with no differences observed in the proportion of those accessing government funding for disability support between the intervention and active control groups. Support provided by participants included practical support (eg, cooking and cleaning; 169/183, 92.3%), nursing (eg, washing/dressing care recipient; reported by 77.9%), and emotional support (eg, talking to the care recipient about their problems; 178/183, 97.2%). Between groups, a greater proportion of participants in the intervention group reported providing nursing support to their care recipients than those in the active control group (χ^2^_1_=6.4; *P*=.01). For 65.1% (119/183) of the participants, no other person was providing support to their care recipient. Furthermore, 97.2% (178/183) of the participants reported that caring for their care recipient had adversely affected the amount of time they were able to spend on themselves. The 2 groups did not differ in either of these factors.

Compared with national caregiver data available from the ABS [2], this study’s sample included a lower proportion of caregivers who were male (current sample: 4.4% and ABS SDAC: 45%), of younger age (current sample mean age: 39.5 years and ABS SDAC mean age: 55 years), and less likely to provide care to a spouse (current sample: 6.0% and ABS SDAC: 40.0%).

**Table 1:** Demographic characteristics of participants from the intervention and active control groups.

Variables		Intervention (n=73)	Active control (n=110)	Group differences			
				*t* test (df)		Chi-square (df)	*P* value
Age (years), mean (SD)		40.29 (6.51)	39.21 (5.86)	1.16 (179)			.25
**Sex, n (%)**				N/A^a^		1.52 (2)	.47
	Male	3 (4)	5 (5)				
	Female	69 (95)	104 (95.4)				
	Other	1 (1)	0 (0)				
**Household income, Aus $ (US** $**), n (%)**				N/A		8.90 (5)	.11
	<15,000 (10,385)	5 (7)	5 (45)				
	15,000-30,000 (10,385-20,771)	19 (26)	19 (17)				
	31,000-60,000 (21,463-41,542)	16 (22)	16 (15)				
	61,000-100,000 (42,235-69,237)	34 (47)	34 (31)				
	101,000-150,000 (69,929-103,856)	21 (29)	21 (19)				
	>150,000 (103,856)	15 (21)	15 (14)				
**Employment status, n (%)**				N/A			
	Full-time paid	11 (15)	15 (14)			0.07 (1)	.79
	Full-time study	9 (12)	10 (9)			0.49 (1)	.48
	Full-time home	31 (43)	35 (32)			2.16 (1)	.14
	Part-time paid	21 (29)	38 (34)			0.67 (1)	.41
	Casual paid	3 (4)	10 (9)			1.65 (1)	.20
	Part-time home	19 (26)	22 (20)			0.92 (1)	.34
	Unemployed	5 (7)	8 (7)			0.01 (1)	.91
Number of care recipients, n (%)		1.66 (0.82)	1.49 (0.71)	N/A		1.50 (182)	.13
**Primary care recipient, n (%)**				N/A		3.17 (4)	.53
	Parent	5 (7)	6 (5)				
	Spouse	5 (7)	8 (7)				
	Child	60 (82)	85 (77)				
	Friend	2 (3)	5 (5)				
	Other	1 (1)	7 (6)				
**Care burden (hours per week), n (%)**				N/A		4.31 (3)	.23
	<20	4 (6)	12 (12)				
	20-29	3 (4)	11 (11)				
	30-39	3 (4)	5 (5)				
	>40	59 (86)	76 (73)				
**Care recipient disability type, n (%)**				N/A			
	Sensory	22 (42)	30 (48)			0.32 (1)	.57
	Intellectual	36 (69)	43 (68)			0.01 (1)	.91
	Physical	25 (48)	25 (40)			0.82 (1)	.37
	Psychosocial	46 (88)	58 (92)			0.43 (1)	.51
	Head injury/stroke or acquired brain injury	1 (2)	3 (5)			0.68 (1)	.41
	Other	16 (31)	25 (40)			0.99 (1)	.32

^a^N/A: not applicable.

### Hypothesis Testing

Table 2 provides the means and SDs for the study variables by group and time points. In general, participants in the intervention group exhibited improvement from baseline to postintervention on a number of (but not all) study variables.

**Table 2:** Descriptive statistics by group and time point for primary and secondary outcomes.

Variables			Active control, mean (SD)				Intervention, mean (SD)				
			Baseline		Postintervention		Baseline		Postintervention		Follow-up
**Primary outcomes**											
	Stress	18.82 (7.98)		18.94 (9.03)		17.03 (7.88)		14.72 (7.49)		12.79 (7.58)	
	Anxiety	8.14 (6.76)		8.61 (6.90)		7.56 (7.60)		6.11 (5.86)		5.58 (5.81)	
	Depression	10.95 (8.00)		10.87 (8.58)		11.33 (8.67)		9.66 (7.71)		7.14 (6.79)	
	Subjective well-being	58.02 (15.18)		54.72 (17.06)		55.73 (16.15)		57.98 (17.54)		62.82 (17.61)	
**Secondary outcomes**											
	Mood affect	52.92 (15.96)		52.37 (19.35)		55.48 (16.88)		58.11 (15.56)		64.27 (16.06)	
	Optimism	5.74 (2.65)		5.81 (2.89)		6.33 (2.51)		6.60 (2.25)		7.41 (2.22)	
	Primary control	40.41 (9.05)		41.50 (10.38)		40.51 (9.32)		42.75 (7.09)		42.82 (7.62)	
	Secondary control	17.88 (6.96)		15.39 (7.91)		20.80 (6.93)		19.60 (8.89)		19.74 (7.53)	
	Self-esteem	9.27 (2.33)		10.06 (2.36)		9.44 (2.77)		10.15 (2.49)		10.97 (2.32)	
	Support_Family	16.56 (6.61)		17.34 (6.79)		16.39 (6.54)		17.66 (6.21)		19.87 (5.39)	
	Support_Friends	18.62 (5.47)		19.42 (5.94)		17.93 (5.65)		19.68 (5.43)		20.08 (5.81)	
	Support_Others	19.91 (5.89)		21.24 (5.45)		20.04 (5.72)		20.91 (5.89)		23.68 (4.29)	

#### Changes From Baseline to Postintervention (Intervention vs Control Group; Hypotheses 1 and 2)

Multilevel modeling indicated a significant time×group interaction by the postintervention time point for the primary outcomes of anxiety (*b*=−2.030; 95% CI −3.607 to −0.453; *P*=.02), depression (*b*=−1.841; 95% CI −3.569 to −0.113; *P*=.04), stress (*b*=−2.159; 95% CI −4.007 to −0.311; *P*=.03), and subjective well-being (*b*=5.454; 95% CI 2.065 to 8.843; *P*=.008).

These significant interaction effects were followed up with simple effects testing to determine changes in outcomes for the control and intervention groups separately. Stress symptoms were significantly reduced in the intervention group (*b*=−2.070; 95% CI −3.743 to −0.397; *P*=.04; Cohen *d*=0.338) but did not change significantly in the control group (*b*=0.246; 95% CI −1.028 to 1.520; *P*=.75; Cohen *d*=0.043). Improvement in depressive symptoms was borderline significant for the intervention condition (*b*=−1.361; 95% CI −2.752 to 0.030; *P*=.05; Cohen *d*=0.267) but did not significantly change in the control group (*b*=0.427; 95% CI −0.697 to 1.551; *P*=.27; Cohen *d*=0.085). Subjective well-being worsened significantly in the control group (*b*=−3.894; 95% CI −5.920 to −1.868; *P*=.002; Cohen *d*=0.428) but did not significantly change in the intervention condition (*b*=1.501; 95% CI −1.540 to 4.542; *P*=.42; Cohen *d*=0.135). Neither the control group (*b*=1.089; 95% CI −0.064 to 2.242; *P*=.06; Cohen *d*=0.210) nor the intervention group (*b*=−0.921; 95% CI −2.182 to 0.340; *P*=.11; Cohen *d*=0.199) significantly changed in the level of anxiety by postintervention, although their symptom change trended in opposite directions (improvement for the intervention group and worsening for the control group), which accounts for the significant group×time interaction.

Among the secondary outcomes, the group×time interaction was only significant for secondary control (*b*=2.522; 95% CI 0.552 to 4.492; *P*=.02). Post hoc testing revealed a significant reduction in secondary control for the control group (*b*=−2.558; 95% CI −3.786 to −1.330; *P*<.001; Cohen *d*=0.463) but a nonsignificant change in secondary control for the intervention group (*b*=−0.030; 95% CI −1.550 to 1.490; *P*=.97; Cohen *d*=0.005).

#### Changes From Postintervention to 3-Month Follow-Up (Intervention Group Only; Hypotheses 3 and 4)

Among the primary outcomes, significant improvements were observed from postintervention to the 3-month follow-up for depression (*b*=−1.824; 95% CI −3.466 to −0.182; *P*=.03; Cohen *d*=0.360) and subjective well-being (*b*=4.825; 95% CI 2.304 to 7.346; *P*<.001; Cohen *d*=0.621) but nonsignificant changes in symptoms of anxiety (*b*=−0.123; 95% CI −1.442 to 1.196; *P*=.86; Cohen *d*=0.030) and stress (*b*=−1.723; 95% CI −3.630 to 0.184; *P*=.08; Cohen *d*=0.293).

Among the secondary outcomes, significant improvements in symptoms were observed for emotional well-being (*b*=6.132; 95% CI 3.451 to 8.813; *P*<.001; Cohen *d*=0.742), optimism (*b*=0.776; 95% CI 0.208 to 1.344; *P*=.007; Cohen *d*=0.443), self-esteem (*b*=−0.842; 95% CI 0.258 to 1.426; *P*=.005; Cohen *d*=0.468), support from family (*b*=2.154; 95% CI 0.872 to 3.436; *P*=.001; Cohen *d*=0.546), and support from significant others (*b*=2.662; 95% CI 1.300 to 4.024; *P*<.001; Cohen *d*=0.634).

#### Modules Completed as Moderator (Hypothesis 5)

In total, 58 of the 73 individuals allocated to the intervention arm viewed at least one module, although all 73 individuals were retained for analyses consistent with the principles of ITT. On average, participants in the intervention condition completed 2.55 out of the 5 modules (SD 1.05). Psychoeducation (56/58, 97%) and values modules (52/58, 90%) were the most commonly used modules, with less viewing of mindfulness (17/58, 29%), well-being (12/58, 21%), and behavioral activation modules (11/58, 19%).

The number of modules completed moderated the level of improvement in primary control from baseline to postintervention for the intervention group (*b*=1.420; 95% CI 0.422 to 2.418; *P*=.01; Cohen *d*=0.389), such that primary control improved further with every additional module completed. The number of modules completed did not moderate any of the other studied variables (all remaining *P* values were >.05 and Cohen *d* values<0.24).

#### User Feedback (Hypothesis 6)

The overall quality of the app was rated highly, with a mean score of 3.94 out of a maximum score of 5 (SD 0.58). Participants rated their subjective quality of the app slightly lower (mean 3.19, SD 0.85). Within the subjective quality subscale, participants expressed that they would not choose to pay for the app (mean 2.22, SD 1.14), which was the only item to be rated with a mean score below 2.5. The app was rated particularly positively for its functionality (mean 4.19, SD 0.75), information (mean 3.96, SD 0.63), and aesthetics (mean 3.95, SD 0.63). Although all subscales were rated highly, the engagement subscale achieved the lowest mean score (mean 3.68, SD 0.65). Within the engagement subscale, the items assessing customization and interactivity were rated the lowest (mean 3.31, SD 0.85; and mean 3.47, SD 0.82, respectively).

## Discussion

### Principal Findings

The purpose of this study was to evaluate the efficacy of a mobile app–based, self-directed psychological intervention for individuals providing care to family or friends with a physical or mental condition. The sample predominantly consisted of mothers of children with a disability with high levels of care burden and stress. The intervention group experienced improvements in the primary outcomes of stress, depression, anxiety, and subjective well-being across the intervention period despite using only a small number of the treatment modules offered, with further improvements in mental health and outlook observed over the 3- to 4-month follow-up period. Participants rated the intervention app highly for its usability and quality, with the potential to improve the app design further through the addition of greater personalization and flexibility. Given the limited number of studies that have investigated the potential of mobile health (mHealth) tools for caregiver populations, the results of this study have important implications for future work in this field.

We found that caregivers initially presented with challenging caring contexts and elevated levels of distress. Importantly, the study sample differed in several ways from national survey data on caregivers in Australia (collected by the SDAC [2]). Notably, participants in this study were more likely to be female, of younger age, experiencing a high care burden, and more likely to be caring for children with a disability, compared with participants in the SDAC study. Participant recruitment was extended to caregivers of all demographic types, suggesting that a self-selection bias occurred favoring a specific caregiving context. Differences between this study’s sample and the SDAC sample may indicate that younger female caregivers may be more help seeking and have greater familiarity with, or interest in, seeking help through technologies, such as mHealth, social media, and/or other digital health interventions, compared with caregivers more broadly [78]. Furthermore, the higher prevalence of parents of children with disabilities in this study’s sample than in the SDAC may suggest that the concept of an app-based mental health intervention has particular applicability to this caregiving context. A significant body of literature has shown that parents of children with disabilities, particularly autism spectrum disorder, experience highly elevated levels of depression and stress [79,80], with very few interventions targeting the mental health needs of this population and fewer again being offered through digital platforms [81]. Digital health initiatives may be particularly appealing to this demographic of caregivers, given the high levels of need, convenience, flexibility, and speed offered [82,83]. The high proportion of younger women caring for children in this study’s sample means that some caution is needed when generalizing our findings to caregivers more broadly. Further research with different subsets of caregivers (such as male caregivers; older caregivers; or those caring for spouses/partners, parents, or siblings) will help clarify the benefits of the StressLess app for these groups.

Intervention-related effects were observed despite the somewhat low usage across intervention modules. Although participants tended to not complete all modules provided by the app, the modules participants chose to complete appear to have been effective. This finding is consistent with the broader literature, which has found that the therapeutic techniques presented in each module are independently associated with improvements in mental health and well-being [27,28]. Participants in this study appear to have targeted their use to specific modules, which may be reflective of their high stress and time-limited context [84]. This finding suggests that flexible intervention designs may be particularly important with caregiver populations as they enable individuals to tailor programs to their needs. Structured mHealth interventions that require high levels of compliance from participants may not show the same levels of improvement as observed in this study, given the difficulties caregivers face in managing competing demands [84]. Further exploration of ways to reduce intervention-related workload while ensuring positive outcomes is needed. Testing the modules that are most efficacious may provide data for the StressLess app to recommend specific combinations of modules as most important. Augmenting longer modules, as per the StressLess app, with microintervention content, may also help to provide immediate symptom relief when needed, but without unrealistic time commitments [76].

Although participants rated the quality of the StressLess intervention app highly, their feedback expressed a desire for greater personalization and flexibility in the app design. This suggests that caregivers may benefit from greater opportunities for customization and interactivity in the intervention app’s user experience. Evidence from the broader mHealth literature indicates that tailoring the user’s experience through personalized feedback, prompting, alerts, and reminders is more effective than providing static content to all users [85,86]. Future research could consider the utility of providing a tailored experience to app users, such that the program dynamically adapts to a participant’s context and usage. On the basis of mood assessments within the app and automatically detected usage patterns, the app could in the future send push notifications with recommendations to engage in specific modules at a given point in time [87,88]. Such tailoring, based on knowledge of a user’s past behavior, is common in consumer apps (eg, Netflix) and may provide similar benefits to users of mHealth interventions by providing support at the time of need, based on previous usage behavior [89,90]. Such systems would benefit from a participatory design to ensure that the intelligent health system adequately balances automated decision making with the user’s own input and that its design is also in awareness of privacy concerns that participants may have in disclosing personal data. Developing intelligent and adaptive mHealth interventions through emerging big data technologies, such as machine learning, may be a promising avenue for future research with this population [89,91-93]. This active prompting may also better approximate the structure and support provided in face-to-face therapy.

### Limitations

This study has several limitations. First, as noted earlier, the sample is not broadly representative of caregivers in national studies [2]. This sampling bias may reflect the recruitment and intervention delivery methods used in this study through technology, such as social media and smartphones. Therefore, the results may not be generalizable to other caregiver contexts, particularly to caregivers who face barriers in accessing technology. Future research could aim to examine the effectiveness of mHealth interventions within different caring contexts. Second, given the nature of the intervention, blinding to condition was not possible. This may have impacted the results, as participants could reasonably predict the researchers’ hypotheses. Third, as the study was limited to individuals with an iOS-based phone, it is unclear whether usage patterns and user experiences will generalize to Android users. In Australia, market share is reasonably even for iOS- and Android-based smartphones [94,95], but this is not the case globally. At the very least, this impedes the uptake of the StressLess intervention. It may also signal different demographics that may relate to efficacy, an issue that needs further exploration in eHealth interventions. Fourth, although sample sizes were adequate as calculated through a priori power analysis, attrition across the study duration resulted in smaller numbers in the final assessment. Despite this, for the group attained, moderate effect sizes were identified in the final follow-up. We also note that for ethical reasons and given that the primary analyses were based on the postintervention time point, participants initially assigned to the waitlist control group were granted access to StressLess after the postintervention time point rather than the 3- to 4-month follow-up. As such, stability in outcome measures for the intervention group cannot be compared against changes that would occur for this period without intervention. Finally, the majority of measures used were completed by self-report, with no objective clinical measures of the primary and secondary intervention outcomes of stress, depression, anxiety, and subjective well-being. Nevertheless, both the Depression Anxiety and Stress Scale-21 and PWI have demonstrated sound psychometric properties [60,61] and have been applied in caregiver contexts in previous studies [76].

### Conclusions

Overall, this study has important clinical implications for the design and effective treatment of mHealth interventions for caregivers experiencing stress. First, the results confirm prior studies showing that caregivers commonly report a need for support for their mental health and well-being, particularly in contexts with high levels of care burden [84]. This study’s sample primarily consisted of mothers caring for children with a disability with high levels of care burden and stress, consistent with the broader disability literature [22,96,97]. Second, caregivers experienced improvements in their mental health and well-being despite using only a small number of the modules offered, indicating that burdensome treatment designs may not be necessary in the caregiver context. Finally, caregivers expressed a preference for interventions that are personalized and flexible in their design, with advances in technology offering the potential for ubiquitous, tailored support. Taken together, the StressLess intervention demonstrates that mHealth apps can successfully improve health and well-being in caregivers, with further work needed to evaluate such interventions in other caregiver groups (ie, older or male caregivers) and to ascertain impacts longer term.

## Appendix

Multimedia Appendix 1 Summary of the contents of the five modules of the StressLess intervention.

Multimedia Appendix 2 Summary of reliability estimates per group over time for outcome variables.

Multimedia Appendix 3 CONSORT-EHEALTH checklist (V. 1.6.1).

## Abbreviations

ABS: Australian Bureau of Statistics
CBT: cognitive behavioral therapy
CONSORT: Consolidated Standards of Reporting Trials
DV: dependent variable
eHealth: electronic health
EMA: ecological momentary assessment
ITT: intention-to-treat
mHealth: mobile health
PSCS: Primary and Secondary Control Scale
PWI: Personal Wellbeing Index
SDAC: Survey of Disability, Aging, and Carers
