# Supplementary material for: Efficacy of a Smartphone App Intervention for Reducing Caregiver Stress: Randomized Controlled Trial
Source: JMIR Ment Health. 2020 Jul 24;7(7):e17541. doi: 10.2196/17541 (PMC7414413; doi:10.2196/17541)
Supplement: Multimedia Appendix 1 [file mental_v7i7e17541_app1.docx]

### Supplementary Table 1 Summary of the contents of the five modules of the StressLess intervention

| Module | **Description** |
| --- | --- |
| 1. Introduction | Overview of the app program, including instructions on how to use the app, as well as psychoeducation about stress reduction and introduction to third wave cognitive behavioural therapies (e.g., acceptance and commitment based therapy (ACT), mindfulness based cognitive behavioural therapy (MCBT), positive psychology and behavioural activation. |
| 1. Values | Identifying core personal values across different life domains (work, relationships, play/leisure and health), evaluating current living according to one’s values, understanding the difference between values and goals, setting goals according to values |
| 1. Mindfulness | Increasing mindful awareness/observation of sensations, thoughts and feelings without judgement and unnecessary attempts to change their frequency and form.  Mindful breathing and progressive muscle relaxation exercises |
| 1. Wellbeing | Positive psychology techniques, such as using gratitude diaries and positive imagery to increase positive affect  Cognitive restructuring to reduce negative affect and increase self-esteem and optimism |
| 1. Behavioural activation | Monitoring daily activities including those reflecting the domains of ‘relationships,’ ‘work,’ ‘play’ and ‘health,’ increasing exposure to pleasant or valued activities, savoring pleasant experiences |
