# Supplementary material for: Efficacy of a Smartphone App Intervention for Reducing Caregiver Stress: Randomized Controlled Trial
Source: JMIR Ment Health. 2020 Jul 24;7(7):e17541. doi: 10.2196/17541 (PMC7414413; doi:10.2196/17541)
Supplement: Multimedia Appendix 3 [file mental_v7i7e17541_app3.pdf]

CONSORT-EHEALTH (V 1.6.1) - Submission/Publication Form

The CONSORT-EHEALTH checklist is intended for authors of randomized trials evaluating web-based and Internet-based applications/interventions, including mobile interventions, electronic games (incl multiplayer games), social media, certain telehealth applications, and other interactive and/or networked electronic applications. Some of the items (e.g. all subitems under item 5 - description of the intervention) may also be applicable for other study designs.

The goal of the CONSORT EHEALTH checklist and guideline is to be
a) a guide for reporting for authors of RCTs,
b) to form a basis for appraisal of an ehealth trial (in terms of validity)

CONSORT-EHEALTH items/subitems are MANDATORY reporting items for studies published in the Journal of Medical Internet Research and other journals / scientific societies endorsing the checklist.

Items numbered 1., 2., 3., 4a., 4b etc are original CONSORT or CONSORT-NPT (non-pharmacologic treatment) items.
Items with Roman numerals (i., ii, iii, iv etc.) are CONSORT-EHEALTH extensions/clarifications.

As the CONSORT-EHEALTH checklist is still considered in a formative stage, we would ask that you also RATE ON A SCALE OF 1-5 how important/useful you feel each item is FOR THE PURPOSE OF THE CHECKLIST and reporting guideline (optional).

Mandatory reporting items are marked with a red *.
In the textboxes, either copy & paste the relevant sections from your manuscript into this form - please include any quotes from your manuscript in QUOTATION MARKS,
or answer directly by providing additional information not in the manuscript, or elaborating on why the item was not relevant for this study.

YOUR ANSWERS WILL BE PUBLISHED AS A SUPPLEMENTARY FILE TO YOUR PUBLICATION IN JMIR AND ARE CONSIDERED PART OF YOUR PUBLICATION (IF ACCEPTED).
Please fill in these questions diligently. Information will not be copyedited, so please use proper spelling and grammar, use correct capitalization, and avoid abbreviations.

DO NOT FORGET TO SAVE AS PDF _AND_ CLICK THE SUBMIT BUTTON SO YOUR ANSWERS ARE IN OUR DATABASE !!!

Citation Suggestion (if you append the pdf as Appendix we suggest to cite this paper in the

caption):
Eysenbach G, CONSORT-EHEALTH Group
CONSORT-EHEALTH: Improving and Standardizing Evaluation Reports of Web-based and Mobile Health Interventions
J Med Internet Res 2011;13(4):e126
URL: http://www.jmir.org/2011/4/e126/
doi: 10.2196/jmir.1923
PMID: 22209829

* Required

Your name *

First Last

Matthew Fuller-Tyszkiewicz

Primary Affiliation (short), City, Country *

University of Toronto, Toronto, Canada

Deakin University, Geelong, Australia

Your e-mail address *

abc@gmail.com

matthewf@deakin.edu.au

Title of your manuscript *

Provide the (draft) title of your manuscript.

Efficacy of a Smartphone app intervention for reducing caregiver stress: A randomized controlled trial.

Name of your App/Software/Intervention *

If there is a short and a long/alternate name, write the short name first and add the long name in brackets.

StressLess

Evaluated Version (if any)

e.g. "V1", "Release 2017-03-01", "Version 2.0.27913"

V1

Language(s) *

What language is the intervention/app in? If multiple languages are available, separate by comma (e.g. "English, French")

English

URL of your Intervention Website or App

e.g. a direct link to the mobile app on app in appstore (itunes, Google Play), or URL of the website. If the intervention is a DVD or hardware, you can also link to an Amazon page.

https://apps.apple.com/au/app/stressless/id1

URL of an image/screenshot (optional)

Your answer

Accessibility *

Can an enduser access the intervention presently?

- (●) access is free and open
- ( ) access only for special usergroups, not open
- ( ) access is open to everyone, but requires payment/subscription/in-app purchases
- ( ) app/intervention no longer accessible
- ( ) Other:

Primary Medical Indication/Disease/Condition *

e.g. "Stress", "Diabetes", or define the target group in brackets after the condition, e.g. "Autism (Parents of children with)", "Alzheimers (Informal Caregivers of)"

Informal carers

Primary Outcomes measured in trial *

comma-separated list of primary outcomes reported in the trial

depression, anxiety, stress, subjective wellbein

Secondary/other outcomes

Are there any other outcomes the intervention is expected to affect?

emotional wellbeing, self-esteem, optimism, primary and secondary control, perceived social support

Recommended "Dose" *

What do the instructions for users say on how often the app should be used?

- ( ) Approximately Daily
- (x) Approximately Weekly
- ( ) Approximately Monthly
- ( ) Approximately Yearly
- ( ) "as needed"
- ( ) Other:

Approx. Percentage of Users (starters) still using the app as recommended after 3 months *

- (selected) unknown / not evaluated
- 0-10%
- 11-20%
- 21-30%
- 31-40%
- 41-50%
- 51-60%
- 61-70%
- 71%-80%
- 81-90%
- 91-100%
- Other:

Overall, was the app/intervention effective? *

- [x] yes: all primary outcomes were significantly better in intervention group vs control
- [ ] partly: SOME primary outcomes were significantly better in intervention group vs control
- [ ] no statistically significant difference between control and intervention
- [ ] potentially harmful: control was significantly better than intervention in one or more outcomes
- [ ] inconclusive: more research is needed
- [ ] Other:

Article Preparation Status/Stage *

At which stage in your article preparation are you currently (at the time you fill in this form)

- [ ] not submitted yet - in early draft status
- [ ] not submitted yet - in late draft status, just before submission
- [ ] submitted to a journal but not reviewed yet
- [x] submitted to a journal and after receiving initial reviewer comments
- [ ] submitted to a journal and accepted, but not published yet
- [ ] published
- [ ] Other:

Journal *

If you already know where you will submit this paper (or if it is already submitted), please provide the journal name (if it is not JMIR, provide the journal name under "other")

- ○ not submitted yet / unclear where I will submit this
- ○ Journal of Medical Internet Research (JMIR)
- ○ JMIR mHealth and UHealth
- ○ JMIR Serious Games
- ◉ JMIR Mental Health
- ○ JMIR Public Health
- ○ JMIR Formative Research
- ○ Other JMIR sister journal
- ○ Other:

Is this a full powered effectiveness trial or a pilot/feasibility trial? *

- ○ Pilot/feasibility
- ◉ Fully powered

Manuscript tracking number *

If this is a JMIR submission, please provide the manuscript tracking number under "other" (The ms tracking number can be found in the submission acknowledgement email, or when you login as author in JMIR. If the paper is already published in JMIR, then the ms tracking number is the four-digit number at the end of the DOI, to be found at the bottom of each published article in JMIR)

- ○ no ms number (yet) / not (yet) submitted to / published in JMIR
- ◉ Other: JMH ms#17541

TITLE AND ABSTRACT

1a) TITLE: Identification as a randomized trial in the title

1a) Does your paper address CONSORT item 1a? *

I.e does the title contain the phrase "Randomized Controlled Trial"? (if not, explain the reason under "other")

- ◉ yes
- ○ Other:

1a-i) Identify the mode of delivery in the title

Identify the mode of delivery. Preferably use “web-based” and/or “mobile” and/or “electronic game” in the title. Avoid ambiguous terms like “online”, “virtual”, “interactive”. Use “Internet-based” only if Intervention includes non-web-based Internet components (e.g. email), use “computer-based” or “electronic” only if offline products are used. Use “virtual” only in the context of “virtual reality” (3-D worlds). Use “online” only in the context of “online support groups”. Complement or substitute product names with broader terms for the class of products (such as “mobile” or “smart phone” instead of “iphone”), especially if the application runs on different platforms.

	1	2	3	4	5	
subitem not at all important	○	○	○	○	◉	essential

Does your paper address subitem 1a-i? *

Copy and paste relevant sections from manuscript title (include quotes in quotation marks "like this" to indicate direct quotes from your manuscript), or elaborate on this item by providing additional information not in the ms, or briefly explain why the item is not applicable/relevant for your study

Efficacy of a "Smartphone app intervention" for reducing caregiver stress: A randomized controlled trial.

1a-ii) Non-web-based components or important co-interventions in title

Mention non-web-based components or important co-interventions in title, if any (e.g., “with telephone support”).

	1	2	3	4	5	
subitem not at all important	◉	○	○	○	○	essential

Does your paper address subitem 1a-ii?

Copy and paste relevant sections from manuscript title (include quotes in quotation marks "like this" to indicate direct quotes from your manuscript), or elaborate on this item by providing additional information not in the ms, or briefly explain why the item is not applicable/relevant for your study

n/a - we do not have telephone or other support

1a-iii) Primary condition or target group in the title

Mention primary condition or target group in the title, if any (e.g., “for children with Type I Diabetes”) Example: A Web-based and Mobile Intervention with Telephone Support for Children with Type I Diabetes: Randomized Controlled Trial

	1	2	3	4	5	
subitem not at all important	○	○	●	○	○	essential

Does your paper address subitem 1a-iii? *

Copy and paste relevant sections from manuscript title (include quotes in quotation marks "like this" to indicate direct quotes from your manuscript), or elaborate on this item by providing additional information not in the ms, or briefly explain why the item is not applicable/relevant for your study

Efficacy of a Smartphone app intervention for reducing "caregiver" stress: A randomized controlled trial.

1b) ABSTRACT: Structured summary of trial design, methods, results, and conclusions

NPT extension: Description of experimental treatment, comparator, care providers, centers, and blinding status.

1b-i) Key features/functionalities/components of the intervention and comparator in the METHODS section of the ABSTRACT

Mention key features/functionalities/components of the intervention and comparator in the abstract. If possible, also mention theories and principles used for designing the site. Keep in mind the needs of systematic reviewers and indexers by including important synonyms. (Note: Only report in the abstract what the main paper is reporting. If this information is missing from the main body of text, consider adding it)

	1	2	3	4	5	
subitem not at all important	○	○	◉	○	○	essential

Does your paper address subitem 1b-i? *

Copy and paste relevant sections from the manuscript abstract (include quotes in quotation marks "like this" to indicate direct quotes from your manuscript), or elaborate on this item by providing additional information not in the ms, or briefly explain why the item is not applicable/relevant for your study

"The intervention app contained treatment modules combining daily self-monitoring with third-wave (mindfulness based) cognitive behavioural therapies, while the active control app contained the self-monitoring feature only. Both programs were completed over a 5-week period"

1b-ii) Level of human involvement in the METHODS section of the ABSTRACT

Clarify the level of human involvement in the abstract, e.g., use phrases like “fully automated” vs. “therapist/nurse/care provider/physician-assisted” (mention number and expertise of providers involved, if any). (Note: Only report in the abstract what the main paper is reporting. If this information is missing from the main body of text, consider adding it)

	1	2	3	4	5	
subitem not at all important	○	○	◉	○	○	essential

Does your paper address subitem 1b-ii?

Copy and paste relevant sections from the manuscript abstract (include quotes in quotation marks "like this" to indicate direct quotes from your manuscript), or elaborate on this item by providing additional information not in the ms, or briefly explain why the item is not applicable/relevant for your study

This study aimed to evaluate the effectiveness of a "self-guided mobile app-based psychological intervention"

1b-iii) Open vs. closed, web-based (self-assessment) vs. face-to-face assessments in the METHODS section of the ABSTRACT

Mention how participants were recruited (online vs. offline), e.g., from an open access website or from a clinic or a closed online user group (closed usergroup trial), and clarify if this was a purely web-based trial, or there were face-to-face components (as part of the intervention or for assessment). Clearly say if outcomes were self-assessed through questionnaires (as common in web-based trials). Note: In traditional offline trials, an open trial (open-label trial) is a type of clinical trial in which both the researchers and participants know which treatment is being administered. To avoid confusion, use “blinded” or “unblinded” to indicated the level of blinding instead of “open”, as “open” in web-based trials usually refers to “open access” (i.e. participants can self-enrol). (Note: Only report in the abstract what the main paper is reporting. If this information is missing from the main body of text, consider adding it)

	1	2	3	4	5	
subitem not at all important	○	○	◉	○	○	essential

Does your paper address subitem 1b-iii?

Copy and paste relevant sections from the manuscript abstract (include quotes in quotation marks "like this" to indicate direct quotes from your manuscript), or elaborate on this item by providing additional information not in the ms, or briefly explain why the item is not applicable/relevant for your study

In a randomised single-blind controlled trial, 183 caregivers "recruited online" were randomly allocated to

1b-iv) RESULTS section in abstract must contain use data

Report number of participants enrolled/assessed in each group, the use/uptake of the intervention (e.g., attrition/adherence metrics, use over time, number of logins etc.), in addition to primary/secondary outcomes. (Note: Only report in the abstract what the main paper is reporting. If this information is missing from the main body of text, consider adding it)

	1	2	3	4	5	
subitem not at all important	○	○	○	◉	○	essential

Does your paper address subitem 1b-iv?

Copy and paste relevant sections from the manuscript abstract (include quotes in quotation marks "like this" to indicate direct quotes from your manuscript), or elaborate on this item by providing additional information not in the ms, or briefly explain why the item is not applicable/relevant for your study

Results subsection of Abstract: "In total, 25% of intervention participants were lost to follow-up at 3-months, and 31% of the waitlist control group dropped out before the post-intervention survey... On average, participants completed 2.5 out of 5 treatment modules. The overall quality of the app was also rated highly, with a mean score of 3.94 out of a maximum score of 5."

1b-v) CONCLUSIONS/DISCUSSION in abstract for negative trials

Conclusions/Discussions in abstract for negative trials: Discuss the primary outcome - if the trial is negative (primary outcome not changed), and the intervention was not used, discuss whether negative results are attributable to lack of uptake and discuss reasons. (Note: Only report in the abstract what the main paper is reporting. If this information is missing from the main body of text, consider adding it)

	1	2	3	4	5	
subitem not at all important	○	○	○	◉	○	essential

Does your paper address subitem 1b-v?

Copy and paste relevant sections from the manuscript abstract (include quotes in quotation marks "like this" to indicate direct quotes from your manuscript), or elaborate on this item by providing additional information not in the ms, or briefly explain why the item is not applicable/relevant for your study

"Conclusions
This study demonstrates that mHealth psychological interventions are an effective treatment option for caregivers experiencing high levels of stress. Recommendations for improving mHealth interventions for caregivers include offering flexibility and customisation in the treatment design."

INTRODUCTION

2a) In INTRODUCTION: Scientific background and explanation of rationale

2a-i) Problem and the type of system/solution

Describe the problem and the type of system/solution that is object of the study: intended as stand-alone intervention vs. incorporated in broader health care program? Intended for a particular patient population? Goals of the intervention, e.g., being more cost-effective to other interventions, replace or complement other solutions? (Note: Details about the intervention are provided in “Methods” under 5)

	1	2	3	4	5	
subitem not at all important	○	○	○	○	◉	essential

Does your paper address subitem 2a-i? *

Copy and paste relevant sections from the manuscript (include quotes in quotation marks "like this" to indicate direct quotes from your manuscript), or elaborate on this item by providing additional information not in the ms, or briefly explain why the item is not applicable/relevant for your study

"Given the available evidence on the significance of caregiver burden, tailored interventions designed to reduce stress and promote wellbeing in carers are critically important...

[C]aregivers face a number of barriers to accessing in-person treatment programs, including economic, geographic and/or mobility factors, limited time to engage in interventions, as well as difficulties finding and/or affording the cost of suitable alternate caregiver support to attend treatment [38–40]. Further to this, caregivers often report difficulties prioritising their own needs or setting aside time for 'non-essential' activities, which may include treatment interventions [41]. Digital technologies may be helpful in addressing issues of accessibility to treatment, particularly when there are barriers to attending the more traditional face-to-face individual or group interventions."

2a-ii) Scientific background, rationale: What is known about the (type of) system

Scientific background, rationale: What is known about the (type of) system that is the object of the study (be sure to discuss the use of similar systems for other conditions/diagnoses, if appropiate), motivation for the study, i.e. what are the reasons for and what is the context for this specific study, from which stakeholder viewpoint is the study performed, potential impact of findings [2]. Briefly justify the choice of the comparator.

	1	2	3	4	5	
subitem not at all important	○	○	○	○	◉	essential

Does your paper address subitem 2a-ii? *

Copy and paste relevant sections from the manuscript (include quotes in quotation marks "like this" to indicate direct quotes from your manuscript), or elaborate on this item by providing additional information not in the ms, or briefly explain why the item is not applicable/relevant for your study

Salient aspect from Introduction: "A number of interventions have successfully adapted CBT techniques to digital platforms for carers using video-teleconferencing, websites with text and/or web-based video education and coaching, and online discussion group technologies. Mobile-app based interventions are notably absent from the caregiver intervention literature, with research needed to examine whether brief interventions, delivered through a mobile phone, can realistically deliver a usable service to caregivers."

The Introduction also includes more detailed background information, demonstrating that carers have poor mental health, are time poor, and benefit from intervention, if they are able to receive treatment.

2b) In INTRODUCTION: Specific objectives or hypotheses

Does your paper address CONSORT subitem 2b? *

Copy and paste relevant sections from the manuscript (include quotes in quotation marks "like this" to indicate direct quotes from your manuscript), or elaborate on this item by providing additional information not in the ms, or briefly explain why the item is not applicable/relevant for your study

"Aims and hypotheses
This study is a randomised controlled trial of a mobile app-based, self-directed psychological intervention for people who are providing care to family or friends with a physical and/or mental disability. It was hypothesised that the intervention would produce a greater reduction in stress, depression and anxiety, as well as increased wellbeing, compared to control participants (Hypothesis 1). To assess the broader impact, we also evaluated emotional wellbeing, self-esteem, optimism, primary and secondary control, and perceived social support (secondary outcomes)(Hypothesis 2).We hypothesised that these improvements in self-reports will be maintained for 3-months post-intervention for primary outcomes (Hypotheses 3) and secondary outcomes (Hypotheses 4). Although the intervention was designed to provide a range of modules with different techniques that could each be useful for improving outcomes, we tested the possibility that the effect of intervention allocation was moderated by amount of treatment modules completed. In particular, we predicted that improvements in primary and secondary outcomes would be stronger for individuals allocated to the treatment condition who engage in more modules (Hypothesis 5). We also explored the usefulness of this form of intervention through caregivers’ perceptions of the app’s engagement, functionality, aesthetics, information, and quality, expecting positive ratings across these metrics for the intervention (Hypothesis 6)."

METHODS

3a) Description of trial design (such as parallel, factorial) including allocation ratio

Does your paper address CONSORT subitem 3a? *

Copy and paste relevant sections from the manuscript (include quotes in quotation marks "like this" to indicate direct quotes from your manuscript), or elaborate on this item by providing additional information not in the ms, or briefly explain why the item is not applicable/relevant for your study

"The design of the trial was a 2 (condition: ‘StressLess’ intervention, ‘StressMonitor’ active control) x 3 (occasion: baseline, post-intervention, 4-month follow-up), parallel, single-blind, randomised controlled trial."
And in the Procedure section:
"Participants were then randomly allocated to either the active control or intervention arm using a 3:2 assignment"

3b) Important changes to methods after trial commencement (such as eligibility criteria), with reasons

Does your paper address CONSORT subitem 3b? *

Copy and paste relevant sections from the manuscript (include quotes in quotation marks "like this" to indicate direct quotes from your manuscript), or elaborate on this item by providing additional information not in the ms, or briefly explain why the item is not applicable/relevant for your study

Not applicable as no changes were made.

3b-i) Bug fixes, Downtimes, Content Changes

Bug fixes, Downtimes, Content Changes: ehealth systems are often dynamic systems. A description of changes to methods therefore also includes important changes made on the intervention or comparator during the trial (e.g., major bug fixes or changes in the functionality or content) (5-iii) and other “unexpected events” that may have influenced study design such as staff changes, system failures/downtimes, etc. [2].

	1	2	3	4	5	
subitem not at all important	(●)	○	○	○	○	essential

Does your paper address subitem 3b-i?

Copy and paste relevant sections from the manuscript (include quotes in quotation marks "like this" to indicate direct quotes from your manuscript), or elaborate on this item by providing additional information not in the ms, or briefly explain why the item is not applicable/relevant for your study

Not important for our study as we (thankfully) did not have issues with the app once launched.

4a) Eligibility criteria for participants

Does your paper address CONSORT subitem 4a? *

Copy and paste relevant sections from the manuscript (include quotes in quotation marks "like this" to indicate direct quotes from your manuscript), or elaborate on this item by providing additional information not in the ms, or briefly explain why the item is not applicable/relevant for your study

"To be eligible to join the study, participants were required to be: (1) an Australian resident; (2) aged 18 years or older; (3) fluent in English; (4) helping to support a friend or relative with a physical or mental condition/disability; (5) able to access an iOS mobile phone device (iPhone or iPad) with internet access for the duration of the study; and (6) to not have participated in an eHealth intervention (any technology-based health intervention, including mobile apps) within the previous 6 months. Smartphone app literacy was also a de facto eligibility criterion, but assumed by the participant's willingness to sign up to the study. A CONSORT flow diagram is provided below (Figure 1)."

4a-i) Computer / Internet literacy

Computer / Internet literacy is often an implicit “de facto” eligibility criterion - this should be explicitly clarified.

	1	2	3	4	5	
subitem not at all important	○	○	○	●	○	essential

Does your paper address subitem 4a-i?

Copy and paste relevant sections from the manuscript (include quotes in quotation marks "like this" to indicate direct quotes from your manuscript), or elaborate on this item by providing additional information not in the ms, or briefly explain why the item is not applicable/relevant for your study

We have added a statement to this effect in the Participants section where we describe eligibility criteria:

... Smartphone app literacy was also a de facto eligibility criterion, but assumed by the participant's willingness to sign up to the study"

4a-ii) Open vs. closed, web-based vs. face-to-face assessments:

Open vs. closed, web-based vs. face-to-face assessments: Mention how participants were recruited (online vs. offline), e.g., from an open access website or from a clinic, and clarify if this was a purely web-based trial, or there were face-to-face components (as part of the intervention or for assessment), i.e., to what degree got the study team to know the participant. In online-only trials, clarify if participants were quasi-anonymous and whether having multiple identities was possible or whether technical or logistical measures (e.g., cookies, email confirmation, phone calls) were used to detect/prevent these.

	1	2	3	4	5	
subitem not at all important	○	○	○	◉	○	essential

Does your paper address subitem 4a-ii? *

Copy and paste relevant sections from the manuscript (include quotes in quotation marks "like this" to indicate direct quotes from your manuscript), or elaborate on this item by providing additional information not in the ms, or briefly explain why the item is not applicable/relevant for your study

"StressLess is a five-week, self-directed intervention". As detailed in the Procedure, following randomization: "Participants then completed five weeks with their assigned app, with weekly contact from the research team by either an email or phone call to maximise engagement. In more detail, a standard email was sent to all participants in each group in weeks 1, 2, 4 and 5 explaining an aspect of either the intervention or the active control program.... They were not designed for therapeutic purposes".

4a-iii) Information giving during recruitment

Information given during recruitment. Specify how participants were briefed for recruitment and in the informed consent procedures (e.g., publish the informed consent documentation as appendix, see also item X26), as this information may have an effect on user self-selection, user expectation and may also bias results.

	1	2	3	4	5	
subitem not at all important	○	○	◉	○	○	essential

Does your paper address subitem 4a-iii?

Copy and paste relevant sections from the manuscript (include quotes in quotation marks "like this" to indicate direct quotes from your manuscript), or elaborate on this item by providing additional information not in the ms, or briefly explain why the item is not applicable/relevant for your study

As detailed in the Participants section: "Participants were recruited through a mix of traditional strategies and targeted social media advertising. Support was sought from caregiver organisations and services, who agreed to display study flyers (both in physical and digital forms), and allowed the research team to attend caregiver events and seminars for recruitment purposes. Social media advertising was conducted through Facebook, with separate ad campaigns targeting either Australians broadly or those with an interest in specific disability topics (e.g., “Attention deficit hyperactivity disorder awareness”, “Alzheimer’s awareness”, “physical disability”). Campaigns were restricted to adult Facebook users located in Australia accessing the platform through an iOS device."

4b) Settings and locations where the data were collected

Does your paper address CONSORT subitem 4b? *

Copy and paste relevant sections from the manuscript (include quotes in quotation marks "like this" to indicate direct quotes from your manuscript), or elaborate on this item by providing additional information not in the ms, or briefly explain why the item is not applicable/relevant for your study

All data were collected via Qualtrics (for baseline and follow-up surveys). This is detailed in the Method section:

"After providing written informed consent and meeting the study eligibility criteria, participants were invited to complete the baseline assessment as a web survey... Participants then completed the post-intervention assessment as a web survey."

4b-i) Report if outcomes were (self-)assessed through online questionnaires

Clearly report if outcomes were (self-)assessed through online questionnaires (as common in web-based trials) or otherwise.

	1	2	3	4	5	
subitem not at all important	○	○	○	○	◉	essential

Does your paper address subitem 4b-i? *

Copy and paste relevant sections from the manuscript (include quotes in quotation marks "like this" to indicate direct quotes from your manuscript), or elaborate on this item by providing additional information not in the ms, or briefly explain why the item is not applicable/relevant for your study

"After providing written informed consent and meeting the study eligibility criteria, participants were invited to complete the baseline assessment as a web survey... Participants then completed the post-intervention assessment as a web survey."

4b-ii) Report how institutional affiliations are displayed

Report how institutional affiliations are displayed to potential participants [on ehealth media], as affiliations with prestigious hospitals or universities may affect volunteer rates, use, and reactions with regards to an intervention.(Not a required item – describe only if this may bias results)

	1	2	3	4	5	
subitem not at all important	○	○	○	●	○	essential

Does your paper address subitem 4b-ii?

Copy and paste relevant sections from the manuscript (include quotes in quotation marks "like this" to indicate direct quotes from your manuscript), or elaborate on this item by providing additional information not in the ms, or briefly explain why the item is not applicable/relevant for your study

"Although the advertisements did not immediately identify the institutional affiliations of the research team, this was made clear in the Plain Language Statement that participants were directed to via weblinks to start the program."

5) The interventions for each group with sufficient details to allow replication, including how and when they were actually administered

5-i) Mention names, credential, affiliations of the developers, sponsors, and owners

Mention names, credential, affiliations of the developers, sponsors, and owners [6] (if authors/evaluators are owners or developer of the software, this needs to be declared in a “Conflict of interest” section or mentioned elsewhere in the manuscript).

	1	2	3	4	5	
subitem not at all important	○	○	○	●	○	essential

Does your paper address subitem 5-i?

Copy and paste relevant sections from the manuscript (include quotes in quotation marks "like this" to indicate direct quotes from your manuscript), or elaborate on this item by providing additional information not in the ms, or briefly explain why the item is not applicable/relevant for your study

In the acknowledgements section, we emphasise that the project was sponsored by Australian Unity. We also state: "Although author BR made the app, there are no financial incentives to conflict with aims of this manuscript as we have made the app freely available to the public."

5-ii) Describe the history/development process

Describe the history/development process of the application and previous formative evaluations (e.g., focus groups, usability testing), as these will have an impact on adoption/use rates and help with interpreting results.

	1	2	3	4	5	
subitem not at all important	○	○	○	◉	○	essential

Does your paper address subitem 5-ii?

Copy and paste relevant sections from the manuscript (include quotes in quotation marks "like this" to indicate direct quotes from your manuscript), or elaborate on this item by providing additional information not in the ms, or briefly explain why the item is not applicable/relevant for your study

We do not have a formative evaluation (e.g., focus groups or usability tests) for the app, but made a key focus in the current paper to get feedback from participants about user experience. These results are a stated aim and detailed in Results and Discussion sections.

5-iii) Revisions and updating

Revisions and updating. Clearly mention the date and/or version number of the application/intervention (and comparator, if applicable) evaluated, or describe whether the intervention underwent major changes during the evaluation process, or whether the development and/or content was "frozen" during the trial. Describe dynamic components such as news feeds or changing content which may have an impact on the replicability of the intervention (for unexpected events see item 3b).

	1	2	3	4	5	
subitem not at all important	○	◉	○	○	○	essential

Does your paper address subitem 5-iii?

Copy and paste relevant sections from the manuscript (include quotes in quotation marks "like this" to indicate direct quotes from your manuscript), or elaborate on this item by providing additional information not in the ms, or briefly explain why the item is not applicable/relevant for your study

The app remains in version 1. There have not been any issues with the app, nor revisions made since time of testing.

5-iv) Quality assurance methods

Provide information on quality assurance methods to ensure accuracy and quality of information provided [1], if applicable.

	1	2	3	4	5	
subitem not at all important	○	○	◉	○	○	essential

Does your paper address subitem 5-iv?

Copy and paste relevant sections from the manuscript (include quotes in quotation marks "like this" to indicate direct quotes from your manuscript), or elaborate on this item by providing additional information not in the ms, or briefly explain why the item is not applicable/relevant for your study

Unfortunately, we did not have external verification of participant responses to survey items. Our main measures are self-report as they ask about internal states, and thus this self-report approach is valid for our purposes.

5-v) Ensure replicability by publishing the source code, and/or providing screenshots/screen-capture video, and/or providing flowcharts of the algorithms used

Ensure replicability by publishing the source code, and/or providing screenshots/screen-capture video, and/or providing flowcharts of the algorithms used. Replicability (i.e., other researchers should in principle be able to replicate the study) is a hallmark of scientific reporting.

	1	2	3	4	5	
subitem not at all important	○	○	○	○	◉	essential

Does your paper address subitem 5-v?

Copy and paste relevant sections from the manuscript (include quotes in quotation marks "like this" to indicate direct quotes from your manuscript), or elaborate on this item by providing additional information not in the ms, or briefly explain why the item is not applicable/relevant for your study

Figure 2 in the manuscript provides snapshots of the app. The app is also available on the AppStore for iOS users. We include a note in the Method section about access to the source code: "Source code for StressLess is available upon request to the corresponding author."

5-vi) Digital preservation

Digital preservation: Provide the URL of the application, but as the intervention is likely to change or disappear over the course of the years; also make sure the intervention is archived (Internet Archive, webcitation.org, and/or publishing the source code or screenshots/videos alongside the article). As pages behind login screens cannot be archived, consider creating demo pages which are accessible without login.

	1	2	3	4	5	
subitem not at all important	●	○	○	○	○	essential

Does your paper address subitem 5-vi?

Copy and paste relevant sections from the manuscript (include quotes in quotation marks "like this" to indicate direct quotes from your manuscript), or elaborate on this item by providing additional information not in the ms, or briefly explain why the item is not applicable/relevant for your study

The app is available on the AppStore rather than via a personal website. We also make the source code available upon request to those who wish to see it.

5-vii) Access

Access: Describe how participants accessed the application, in what setting/context, if they had to pay (or were paid) or not, whether they had to be a member of specific group. If known, describe how participants obtained “access to the platform and Internet” [1]. To ensure access for editors/reviewers/readers, consider to provide a “backdoor” login account or demo mode for reviewers/readers to explore the application (also important for archiving purposes, see vi).

	1	2	3	4	5	
subitem not at all important	○	○	○	●	○	essential

Does your paper address subitem 5-vii? *

Copy and paste relevant sections from the manuscript (include quotes in quotation marks "like this" to indicate direct quotes from your manuscript), or elaborate on this item by providing additional information not in the ms, or briefly explain why the item is not applicable/relevant for your study

In the Procedure section, we write: "Instructions were provided to participants detailing how to install the application (either StressLess or StressMonitor) on their mobile phone or iPad. The app is free and does not include any hidden costs." We also emphasise that the app is available from the iOS App Store. (same paragraph)

5-viii) Mode of delivery, features/functionalities/components of the intervention and comparator, and the theoretical framework

Describe mode of delivery, features/functionalities/components of the intervention and comparator, and the theoretical framework [6] used to design them (instructional strategy [1], behaviour change techniques, persuasive features, etc., see e.g., [7, 8] for terminology). This includes an in-depth description of the content (including where it is coming from and who developed it) [1],” whether [and how] it is tailored to individual circumstances and allows users to track their progress and receive feedback” [6]. This also includes a description of communication delivery channels and – if computer-mediated communication is a component – whether communication was synchronous or asynchronous [6]. It also includes information on presentation strategies [1], including page design principles, average amount of text on pages, presence of hyperlinks to other resources, etc. [1].

	1	2	3	4	5	
subitem not at all important	○	○	○	○	◉	essential

Does your paper address subitem 5-viii? *

Copy and paste relevant sections from the manuscript (include quotes in quotation marks "like this" to indicate direct quotes from your manuscript), or elaborate on this item by providing additional information not in the ms, or briefly explain why the item is not applicable/relevant for your study

We detail this in the Method section:
"Intervention: StressLess
StressLess is a five-week, self-directed intervention, based on the principles of second and third wave cognitive behavioural therapies (CBT) [54], delivered through a mobile application (see Figure 2). The intervention provides psycho-education (through text, video, audio, and graphics) and a series of interactive exercises or activities. The intervention comprises five modules (detailed in Supplementary Table 1) as: (1) an introduction involving psychoeducation about stress reduction and third wave CBT; (2) values clarification and goal setting; (3) mindfulness skills involving observation of the self and connection with the present moment, cognitive defusion and acceptance; (4) wellbeing enhancement through positive psychology techniques and cognitive restructuring; and, (5) behavioural activation to increase engagement in, and enjoyment of, pleasant or valued activities. A 'Troubleshooting' tab was also available beyond the core intervention modules, which contained a series of activities to help with stress (e.g., de-stress with a body scan, breathing to diffuse negative thoughts, etc.)."

5-ix) Describe use parameters

Describe use parameters (e.g., intended "doses" and optimal timing for use). Clarify what instructions or recommendations were given to the user, e.g., regarding timing, frequency, heaviness of use, if any, or was the intervention used ad libitum.

	1	2	3	4	5	
subitem not at all important	○	○	○	○	◉	essential

Does your paper address subitem 5-ix?

Copy and paste relevant sections from the manuscript (include quotes in quotation marks "like this" to indicate direct quotes from your manuscript), or elaborate on this item by providing additional information not in the ms, or briefly explain why the item is not applicable/relevant for your study

"StressLess is a five-week, self-directed intervention... The intervention content was designed to provide a suite of therapeutic techniques with demonstrated efficacy in the broader literature, enabling participants' autonomy in selecting the techniques that they feel work best for them. Participants could work through the modules at their own pace and in any order across the five weeks, but were encouraged to complete one module per week in a recommended sequence."

5-x) Clarify the level of human involvement

Clarify the level of human involvement (care providers or health professionals, also technical assistance) in the e-intervention or as co-intervention (detail number and expertise of professionals involved, if any, as well as “type of assistance offered, the timing and frequency of the support, how it is initiated, and the medium by which the assistance is delivered”. It may be necessary to distinguish between the level of human involvement required for the trial, and the level of human involvement required for a routine application outside of a RCT setting (discuss under item 21 – generalizability).

	1	2	3	4	5	
subitem not at all important	○	○	○	○	●	essential

Does your paper address subitem 5-x?

Copy and paste relevant sections from the manuscript (include quotes in quotation marks "like this" to indicate direct quotes from your manuscript), or elaborate on this item by providing additional information not in the ms, or briefly explain why the item is not applicable/relevant for your study

In the Procedure, we emphasise: "Participants then completed five weeks with their assigned app, with weekly contact from the research team by either an email or phone call to maximise engagement. In more detail, a standard email was sent to all participants in each group in weeks 1, 2, 4 and 5 explaining an aspect of either the intervention or the active control program. For example, Week 2 emails were titled “Mindfulness with StressLess” and “Mood Monitoring: How does your mood change across the day?” for the intervention and active control conditions, respectively. Additionally, participants were contacted through a phone call in week 3 to answer any queries about use of the app. These phone calls were to identify any technical difficulities and to maintain engagement. They were not designed for therapeutic purposes."

5-xi) Report any prompts/reminders used

Report any prompts/reminders used: Clarify if there were prompts (letters, emails, phone calls, SMS) to use the application, what triggered them, frequency etc. It may be necessary to distinguish between the level of prompts/reminders required for the trial, and the level of prompts/reminders for a routine application outside of a RCT setting (discuss under item 21 – generalizability).

	1	2	3	4	5	
subitem not at all important	○	○	○	○	◉	essential

Does your paper address subitem 5-xi? *

Copy and paste relevant sections from the manuscript (include quotes in quotation marks "like this" to indicate direct quotes from your manuscript), or elaborate on this item by providing additional information not in the ms, or briefly explain why the item is not applicable/relevant for your study

As per the prior question, we have added the following in the Procedure section:

"Participants then completed five weeks with their assigned app, with weekly contact from the research team by either an email or phone call to maximise engagement. In more detail, a standard email was sent to all participants in each group in weeks 1, 2, 4 and 5 explaining an aspect of either the intervention or the active control program. For example, Week 2 emails were titled “Mindfulness with StressLess” and “Mood Monitoring: How does your mood change across the day?” for the intervention and active control conditions, respectively. Additionally, participants were contacted through a phone call in week 3 to answer any queries about use of the app. These phone calls were to identify any technical difficulities and to maintain engagement. They were not designed for therapeutic purposes."

5-xii) Describe any co-interventions (incl. training/support)

Describe any co-interventions (incl. training/support): Clearly state any interventions that are provided in addition to the targeted eHealth intervention, as ehealth intervention may not be designed as stand-alone intervention. This includes training sessions and support [1]. It may be necessary to distinguish between the level of training required for the trial, and the level of training for a routine application outside of a RCT setting (discuss under item 21 – generalizability.

	1	2	3	4	5	
subitem not at all important	◉	○	○	○	○	essential

Does your paper address subitem 5-xii? *

Copy and paste relevant sections from the manuscript (include quotes in quotation marks "like this" to indicate direct quotes from your manuscript), or elaborate on this item by providing additional information not in the ms, or briefly explain why the item is not applicable/relevant for your study

There is no co-intervention element for this study. The participant experience is as per description in the Procedure section.

6a) Completely defined pre-specified primary and secondary outcome measures, including how and when they were assessed

Does your paper address CONSORT subitem 6a? *

Copy and paste relevant sections from the manuscript (include quotes in quotation marks "like this" to indicate direct quotes from your manuscript), or elaborate on this item by providing additional information not in the ms, or briefly explain why the item is not applicable/relevant for your study

This is detailed throughout the manuscript. The aims/hypotheses section at the end of the Introduction lists each outcome measure, and separates into primary and secondary outcomes. We follow this labelling convention in the Measures section and in reporting and discussion of results.

6a-i) Online questionnaires: describe if they were validated for online use and apply CHERRIES items to describe how the questionnaires were designed/deployed

If outcomes were obtained through online questionnaires, describe if they were validated for online use and apply CHERRIES items to describe how the questionnaires were designed/deployed [9].

	1	2	3	4	5	
subitem not at all important	○	○	●	○	○	essential

Does your paper address subitem 6a-i?

Copy and paste relevant sections from manuscript text

These self-report measures used for primary and secondary outcomes were deployed via Qualtrics, an online survey platform. While we are not aware of specific studies comparing psychometric properties of these measures for online vs face-to-face administration, we wish to emphasise that these are common measures that are typically used in online research. We have added mention of examples of online use of the measures in the Measures section. In each of these cited studies, psychometric properties support their use in online contexts.

6a-ii) Describe whether and how “use” (including intensity of use/dosage) was defined/measured/monitored

Describe whether and how “use” (including intensity of use/dosage) was defined/measured/monitored (logins, logfile analysis, etc.). Use/adoption metrics are important process outcomes that should be reported in any ehealth trial.

	1	2	3	4	5	
subitem not at all important	○	○	○	○	◉	essential

Does your paper address subitem 6a-ii?

Copy and paste relevant sections from manuscript text

We have a dosage-related hypothesis (Hypothesis 6). We also emphasise in the Method section: "Each module that a participant completed was logged by the app to enable tracking of how many modules a participant tried."

6a-iii) Describe whether, how, and when qualitative feedback from participants was obtained

Describe whether, how, and when qualitative feedback from participants was obtained (e.g., through emails, feedback forms, interviews, focus groups).

	1	2	3	4	5	
subitem not at all important	○	○	○	○	◉	essential

Does your paper address subitem 6a-iii?

Copy and paste relevant sections from manuscript text

Feedback on the app was provided at the post-intervention point. This is detailed in the Measures section (what was measured) and the Procedure (timing of measurement).

Measures section:
"App Quality
The intervention App was also assessed for its quality using the Mobile Application Rating Scale [73]. This comprises 23-items rated on a 5-point rating scale. The Mobile Application Rating Scale consists of four subscales: engagement, functionality, aesthetics, and information. The mean item score across the four subscales is used to determine an objective measure of the overall quality of the app, with higher scores indicating higher app quality. Further, the Mobile Application Rating Scale also includes a subscale assessing the subjective quality of the app, consisting of items assessing whether the participant would recommend the app to others, plans to use the app again in the next 12 months, would pay to use the app, and their overall rating of the app out of 5. For the current study, an adapted version of the Mobile Application Rating Scale was used excluding the items assessing the entertainment value and evidence-base for the app. These items were removed from the mean score calculation as per the guidelines [74]."

Procedure:
"The post-intervention survey was identical for participants from both groups, with the exception that the intervention group received the App Quality measure."

6b) Any changes to trial outcomes after the trial commenced, with reasons

Does your paper address CONSORT subitem 6b? *

Copy and paste relevant sections from the manuscript (include quotes in quotation marks "like this" to indicate direct quotes from your manuscript), or elaborate on this item by providing additional information not in the ms, or briefly explain why the item is not applicable/relevant for your study

There were no changes to trial outcomes after the trial commenced

7a) How sample size was determined

NPT: When applicable, details of whether and how the clustering by care provides or centers was addressed

7a-i) Describe whether and how expected attrition was taken into account when calculating the sample size

Describe whether and how expected attrition was taken into account when calculating the sample size.

	1	2	3	4	5	
subitem not at all important	○	○	○	○	●	essential

Does your paper address subitem 7a-i?

Copy and paste relevant sections from manuscript title (include quotes in quotation marks "like this" to indicate direct quotes from your manuscript), or elaborate on this item by providing additional information not in the ms, or briefly explain why the item is not applicable/relevant for your study

From the Method section:

"Sample size calculation
Required sample size was powered with the following assumptions: (1) a moderate group difference (0.5 SD) between the intervention and active control groups for the primary and secondary outcomes at post-intervention; (2) power set at .80; (3) alpha set at .05 (two-tailed); (4) expected attrition rate of 20% for the intervention group [53]; and, (5) an allocation ratio of 3:2 (active control:intervention) under the expectation that attrition would be around 30% for the active control group as they only receive self-monitoring features of the app, and not intervention content during the control phase. Under these assumptions, the adjusted target sample size at baseline was 68 for the intervention and 100 for the active control group."

7b) When applicable, explanation of any interim analyses and stopping guidelines

Does your paper address CONSORT subitem 7b? *

Copy and paste relevant sections from the manuscript (include quotes in quotation marks "like this" to indicate direct quotes from your manuscript), or elaborate on this item by providing additional information not in the ms, or briefly explain why the item is not applicable/relevant for your study

There were no interim analyses and stopping guidelines.

8a) Method used to generate the random allocation sequence

NPT: When applicable, how care providers were allocated to each trial group

Does your paper address CONSORT subitem 8a? *

Copy and paste relevant sections from the manuscript (include quotes in quotation marks "like this" to indicate direct quotes from your manuscript), or elaborate on this item by providing additional information not in the ms, or briefly explain why the item is not applicable/relevant for your study

In the Procedure section, we explain that: "Participants were then randomly allocated to either the active control or intervention arm using a 3:2 assignment in blocks of 5 created through Qualtrics (online survey provider of choice), with the expectation that attrition would be higher in the active control group due to lower incentive to remain in the study."

8b) Type of randomisation; details of any restriction (such as blocking and block size)

Does your paper address CONSORT subitem 8b? *

Copy and paste relevant sections from the manuscript (include quotes in quotation marks "like this" to indicate direct quotes from your manuscript), or elaborate on this item by providing additional information not in the ms, or briefly explain why the item is not applicable/relevant for your study

As per the answer above: "Participants were then randomly allocated to either the active control or intervention arm using a 3:2 assignment in blocks of 5 created through Qualtrics (online survey provider of choice), with the expectation that attrition would be higher in the active control group due to lower incentive to remain in the study."

9) Mechanism used to implement the random allocation sequence (such as sequentially numbered containers), describing any steps taken to conceal the sequence until interventions were assigned

Does your paper address CONSORT subitem 9? *

Copy and paste relevant sections from the manuscript (include quotes in quotation marks "like this" to indicate direct quotes from your manuscript), or elaborate on this item by providing additional information not in the ms, or briefly explain why the item is not applicable/relevant for your study

Randomization was kept separate from the research team by implementing via Qualtrics.

10) Who generated the random allocation sequence, who enrolled participants, and who assigned participants to interventions

Does your paper address CONSORT subitem 10? *

Copy and paste relevant sections from the manuscript (include quotes in quotation marks "like this" to indicate direct quotes from your manuscript), or elaborate on this item by providing additional information not in the ms, or briefly explain why the item is not applicable/relevant for your study

This was generated by Qualtrics.

11a) If done, who was blinded after assignment to interventions (for example, participants, care providers, those assessing outcomes) and how

NPT: Whether or not administering co-interventions were blinded to group assignment

11a-i) Specify who was blinded, and who wasn’t

Specify who was blinded, and who wasn’t. Usually, in web-based trials it is not possible to blind the participants [1, 3] (this should be clearly acknowledged), but it may be possible to blind outcome assessors, those doing data analysis or those administering co-interventions (if any).

	1	2	3	4	5	
subitem not at all important	○	○	○	○	●	essential

Does your paper address subitem 11a-i? *

Copy and paste relevant sections from the manuscript (include quotes in quotation marks "like this" to indicate direct quotes from your manuscript), or elaborate on this item by providing additional information not in the ms, or briefly explain why the item is not applicable/relevant for your study

Blinding was not possible because: (1) participants either received the intervention or an active control, and (2) we needed to know group assignment to provide the appropriate follow-up emails.

11a-ii) Discuss e.g., whether participants knew which intervention was the “intervention of interest” and which one was the “comparator”

Informed consent procedures (4a-ii) can create biases and certain expectations - discuss e.g., whether participants knew which intervention was the “intervention of interest” and which one was the “comparator”.

	1	2	3	4	5	
subitem not at all important	○	○	○	○	◉	essential

Does your paper address subitem 11a-ii?

Copy and paste relevant sections from the manuscript (include quotes in quotation marks "like this" to indicate direct quotes from your manuscript), or elaborate on this item by providing additional information not in the ms, or briefly explain why the item is not applicable/relevant for your study

We emphasise as a limitation: "Second, given the nature of the intervention, blinding to condition was not possible. This may have impacted results as participants could reasonably predict the researchers’ hypotheses."

11b) If relevant, description of the similarity of interventions

(this item is usually not relevant for ehealth trials as it refers to similarity of a placebo or sham intervention to a active medication/intervention)

Does your paper address CONSORT subitem 11b? *

Copy and paste relevant sections from the manuscript (include quotes in quotation marks "like this" to indicate direct quotes from your manuscript), or elaborate on this item by providing additional information not in the ms, or briefly explain why the item is not applicable/relevant for your study

not applicable. we have an intervention condition with the treatment content, and a minimal active condition with simple monitoring.

12a) Statistical methods used to compare groups for primary and secondary outcomes

NPT: When applicable, details of whether and how the clustering by care providers or centers was addressed

Does your paper address CONSORT subitem 12a? *

Copy and paste relevant sections from the manuscript (include quotes in quotation marks "like this" to indicate direct quotes from your manuscript), or elaborate on this item by providing additional information not in the ms, or briefly explain why the item is not applicable/relevant for your study

The Method section details our analytic approach to address primary and secondary outcomes:

"Following the principles of intention-to-treat (ITT) analysis, individuals were retained in the group they were randomized to. Thus, even in cases where participants in the intervention group did not use the app at all (n = 5), they were retained in the intervention group for the purposes of analysis. Missing data were handled using multiple imputation, with 50 imputations. By default, Mplus uses Monte Carlo Markov Chains with 100 iterations per imputation, and chained equations to impute missing values for variables [75]. These imputed files were then imported into Mplus version 8 for multilevel modelling to test: (1) efficacy of the intervention compared to the control condition across study variables at post-intervention for primary outcomes (Hypothesis 1) and secondary outcomes (Hypothesis 2); (2) maintenance of treatment effects at the 4-month follow-up assessment for primary and secondary outcomes (Hypotheses 3 and 4, respectively); and (3) impact of the number of modules completed on treatment efficacy (dose-response effects) (Hypothesis 5).
For the evaluation of efficacy, time was entered as a Level 1 predictor (0 = baseline, 1 = post-intervention). At Level 2, group (0 = control, 1 = intervention) was included as a predictor of the dependent variable (DV) and also as a moderator of the Level 1 relationship between time and DV scores. This latter effect (a cross-level interaction) was used to ascertain whether the rate of improvement in symptoms was greater for intervention participants than those in the control group (Hypotheses 1-2). Maintenance effects were tested similarly, though the time effect compared post-intervention (coded 0) against the 4-month follow-up timepoint (coded 1)(Hypotheses 3-4). As the follow-up data were only collected for the intervention group, there was no Level 2 predictor for group. Dose-response effects were tested with the intervention group only, by moderating the time effect by number of modules completed (Hypothesis 5). Each outcome variable was modelled separately. Descriptive statistics were reported for evaluation of user ratings of the intervention (Hypothesis 6). All effects were tested at $P < .05$ (two-tailed) unless otherwise indicated."

12a-i) Imputation techniques to deal with attrition / missing values

Imputation techniques to deal with attrition / missing values: Not all participants will use the intervention/comparator as intended and attrition is typically high in ehealth trials. Specify how participants who did not use the application or dropped out from the trial were treated in the statistical analysis (a complete case analysis is strongly discouraged, and simple imputation techniques such as LOCF may also be problematic [4]).

	1	2	3	4	5	
subitem not at all important	○	○	○	○	◉	essential

Does your paper address subitem 12a-i? *

Copy and paste relevant sections from the manuscript (include quotes in quotation marks "like this" to indicate direct quotes from your manuscript), or elaborate on this item by providing additional information not in the ms, or briefly explain why the item is not applicable/relevant for your study

"Following the principles of intention-to-treat (ITT) analysis, individuals were retained in the group they were randomized to. Thus, even in cases where participants in the intervention group did not use the app at all (n = 15), they were retained in the intervention group for the purposes of analysis. Missing data were handled using multiple imputation, with 50 imputations. By default, Mplus uses Monte Carlo Markov Chains with 100 iterations per imputation, and chained equations to impute missing values for variables [75]."

12b) Methods for additional analyses, such as subgroup analyses and adjusted analyses

Does your paper address CONSORT subitem 12b? *

Copy and paste relevant sections from the manuscript (include quotes in quotation marks "like this" to indicate direct quotes from your manuscript), or elaborate on this item by providing additional information not in the ms, or briefly explain why the item is not applicable/relevant for your study

We follow up in the intervention group for dose-response effects and also for satisfaction with the intervention:

"Dose-response effects were tested with the intervention group only, by moderating the time effect by number of modules completed (Hypothesis 5). Each outcome variable was modelled separately. Descriptive statistics were reported for evaluation of user ratings of the intervention (Hypothesis 6)."

X26) REB/IRB Approval and Ethical Considerations [recommended as subheading under "Methods"] (not a CONSORT item)

X26-i) Comment on ethics committee approval

	1	2	3	4	5	
subitem not at all important	○	○	○	○	◉	essential

Does your paper address subitem X26-i?

Copy and paste relevant sections from the manuscript (include quotes in quotation marks "like this" to indicate direct quotes from your manuscript), or elaborate on this item by providing additional information not in the ms, or briefly explain why the item is not applicable/relevant for your study

"This study was approved by the Deakin University Human Research Ethics Committee (2016-151)"

x26-ii) Outline informed consent procedures

Outline informed consent procedures e.g., if consent was obtained offline or online (how? Checkbox, etc.?), and what information was provided (see 4a-ii). See [6] for some items to be included in informed consent documents.

	1	2	3	4	5	
subitem not at all important	○	○	○	○	◉	essential

Does your paper address subitem X26-ii?

Copy and paste relevant sections from the manuscript (include quotes in quotation marks "like this" to indicate direct quotes from your manuscript), or elaborate on this item by providing additional information not in the ms, or briefly explain why the item is not applicable/relevant for your study

Procedure section: "After providing informed consent via Qualtrics (by reading a Plain Language Statement and then responding to a question about whether they consented)..."

X26-iii) Safety and security procedures

Safety and security procedures, incl. privacy considerations, and any steps taken to reduce the likelihood or detection of harm (e.g., education and training, availability of a hotline)

	1	2	3	4	5	
subitem not at all important	○	○	○	○	○	essential

Does your paper address subitem X26-iii?

Copy and paste relevant sections from the manuscript (include quotes in quotation marks "like this" to indicate direct quotes from your manuscript), or elaborate on this item by providing additional information not in the ms, or briefly explain why the item is not applicable/relevant for your study

Procedure section: "For both groups, the Plain Language Statement provided via the baseline Qualtrics survey provided contact detail for free helplines should participants feel distressed at any stage due to the intervention. The StressLess and StressMonitor apps also contained these contact details in the app to remind participants that they could contact LifeLine (a free, Australian counselling service) if they felt distressed."

RESULTS

13a) For each group, the numbers of participants who were randomly assigned, received intended treatment, and were analysed for the primary outcome

NPT: The number of care providers or centers performing the intervention in each group and the number of patients treated by each care provider in each center

Does your paper address CONSORT subitem 13a? *

Copy and paste relevant sections from the manuscript (include quotes in quotation marks "like this" to indicate direct quotes from your manuscript), or elaborate on this item by providing additional information not in the ms, or briefly explain why the item is not applicable/relevant for your study

This is detailed in Figure 1, using a CONSORT flow chart.

13b) For each group, losses and exclusions after randomisation, together with reasons

Does your paper address CONSORT subitem 13b? (NOTE: Preferably, this is shown in a CONSORT flow diagram) *

Copy and paste relevant sections from the manuscript (include quotes in quotation marks "like this" to indicate direct quotes from your manuscript), or elaborate on this item by providing additional information not in the ms, or briefly explain why the item is not applicable/relevant for your study

This is detailed in Figure 1, using a CONSORT flow chart.

13b-i) Attrition diagram

Strongly recommended: An attrition diagram (e.g., proportion of participants still logging in or using the intervention/comparator in each group plotted over time, similar to a survival curve) or other figures or tables demonstrating usage/dose/engagement.

	1	2	3	4	5	
subitem not at all important	○	○	○	○	◉	essential

Does your paper address subitem 13b-i?

Copy and paste relevant sections from the manuscript or cite the figure number if applicable (include quotes in quotation marks "like this" to indicate direct quotes from your manuscript), or elaborate on this item by providing additional information not in the ms, or briefly explain why the item is not applicable/relevant for your study

This is detailed in Figure 1, using a CONSORT flow chart.

14a) Dates defining the periods of recruitment and follow-up

Does your paper address CONSORT subitem 14a? *

Copy and paste relevant sections from the manuscript (include quotes in quotation marks "like this" to indicate direct quotes from your manuscript), or elaborate on this item by providing additional information not in the ms, or briefly explain why the item is not applicable/relevant for your study

In the procedure, it is emphasised that the intervention lasts for 5 weeks following baseline assessment, and that there is a 3- to 4-month follow-up assessment for those in the intervention arm.
We also explain in the Participant section: "Recruitment to the baseline component of the study ran from September 2016 to April 2017."

14a-i) Indicate if critical “secular events” fell into the study period

Indicate if critical “secular events” fell into the study period, e.g., significant changes in Internet resources available or “changes in computer hardware or Internet delivery resources”

	1	2	3	4	5	
subitem not at all important	○	○	○	◉	○	essential

Does your paper address subitem 14a-i?

Copy and paste relevant sections from the manuscript (include quotes in quotation marks "like this" to indicate direct quotes from your manuscript), or elaborate on this item by providing additional information not in the ms, or briefly explain why the item is not applicable/relevant for your study

There were no events during the intervention phase to disrupt the study.

14b) Why the trial ended or was stopped (early)

Does your paper address CONSORT subitem 14b? *

Copy and paste relevant sections from the manuscript (include quotes in quotation marks "like this" to indicate direct quotes from your manuscript), or elaborate on this item by providing additional information not in the ms, or briefly explain why the item is not applicable/relevant for your study

not applicable - study did not end early.

15) A table showing baseline demographic and clinical characteristics for each group

NPT: When applicable, a description of care providers (case volume, qualification, expertise, etc.) and centers (volume) in each group

Does your paper address CONSORT subitem 15? *

Copy and paste relevant sections from the manuscript (include quotes in quotation marks "like this" to indicate direct quotes from your manuscript), or elaborate on this item by providing additional information not in the ms, or briefly explain why the item is not applicable/relevant for your study

This is provided in Table 1.

15-i) Report demographics associated with digital divide issues

In ehealth trials it is particularly important to report demographics associated with digital divide issues, such as age, education, gender, social-economic status, computer/Internet/ehealth literacy of the participants, if known.

	1	2	3	4	5	
subitem not at all important	○	○	○	○	◉	essential

Does your paper address subitem 15-i? *

Copy and paste relevant sections from the manuscript (include quotes in quotation marks "like this" to indicate direct quotes from your manuscript), or elaborate on this item by providing additional information not in the ms, or briefly explain why the item is not applicable/relevant for your study

This information is provided in Table 1.

16) For each group, number of participants (denominator) included in each analysis and whether the analysis was by original assigned groups

16-i) Report multiple “denominators” and provide definitions

Report multiple “denominators” and provide definitions: Report N’s (and effect sizes) “across a range of study participation [and use] thresholds” [1], e.g., N exposed, N consented, N used more than x times, N used more than y weeks, N participants “used” the intervention/comparator at specific pre-defined time points of interest (in absolute and relative numbers per group). Always clearly define “use” of the intervention.

	1	2	3	4	5	
subitem not at all important	○	○	○	○	◉	essential

Does your paper address subitem 16-i? *

Copy and paste relevant sections from the manuscript (include quotes in quotation marks "like this" to indicate direct quotes from your manuscript), or elaborate on this item by providing additional information not in the ms, or briefly explain why the item is not applicable/relevant for your study

N is provided in Figure 1. We also emphasise different Ns in the Results section as appropriate: this occurred in one instance, when talking about how many modules were completed (58/73 intervention participants used the app).

16-ii) Primary analysis should be intent-to-treat

Primary analysis should be intent-to-treat, secondary analyses could include comparing only “users”, with the appropriate caveats that this is no longer a randomized sample (see 18-i).

	1	2	3	4	5	
subitem not at all important	○	○	○	○	◉	essential

Does your paper address subitem 16-ii?

Copy and paste relevant sections from the manuscript (include quotes in quotation marks "like this" to indicate direct quotes from your manuscript), or elaborate on this item by providing additional information not in the ms, or briefly explain why the item is not applicable/relevant for your study

"Following the principles of intention-to-treat (ITT) analysis, individuals were retained in the group they were randomized to. Thus, even in cases where participants in the intervention group did not use the app at all (n = 15), they were retained in the intervention group for the purposes of analysis."

17a) For each primary and secondary outcome, results for each group, and the estimated effect size and its precision (such as 95% confidence interval)

Does your paper address CONSORT subitem 17a? *

Copy and paste relevant sections from the manuscript (include quotes in quotation marks "like this" to indicate direct quotes from your manuscript), or elaborate on this item by providing additional information not in the ms, or briefly explain why the item is not applicable/relevant for your study

This is provided in Table 2.

17a-i) Presentation of process outcomes such as metrics of use and intensity of use

In addition to primary/secondary (clinical) outcomes, the presentation of process outcomes such as metrics of use and intensity of use (dose, exposure) and their operational definitions is critical. This does not only refer to metrics of attrition (13-b) (often a binary variable), but also to more continuous exposure metrics such as "average session length". These must be accompanied by a technical description how a metric like a "session" is defined (e.g., timeout after idle time) [1] (report under item 6a).

	1	2	3	4	5	
subitem not at all important	○	○	○	○	◉	essential

Does your paper address subitem 17a-i?

Copy and paste relevant sections from the manuscript (include quotes in quotation marks "like this" to indicate direct quotes from your manuscript), or elaborate on this item by providing additional information not in the ms, or briefly explain why the item is not applicable/relevant for your study

This is provided in the Results section:

"Modules completed as moderator (Hypothesis 5)
In total, 58 of the 73 individuals allocated to the intervention arm viewed at least one module, though all 73 were retained for analyses consistent with principles of ITT. On average, participants in the intervention condition completed 2.55/5 modules (SD = 1.05). The psycho-education (n = 56, 97%) and values modules (n = 52, 90%) were the most commonly used modules, with less viewing of mindfulness (n = 17, 29%), wellbeing (n = 12, 21%), and behavioural activation modules (n = 11, 19%). "

17b) For binary outcomes, presentation of both absolute and relative effect sizes is recommended

Does your paper address CONSORT subitem 17b? *

Copy and paste relevant sections from the manuscript (include quotes in quotation marks "like this" to indicate direct quotes from your manuscript), or elaborate on this item by providing additional information not in the ms, or briefly explain why the item is not applicable/relevant for your study

Not applicable as there were no binary outcomes.

18) Results of any other analyses performed, including subgroup analyses and adjusted analyses, distinguishing pre-specified from exploratory

Does your paper address CONSORT subitem 18? *

Copy and paste relevant sections from the manuscript (include quotes in quotation marks "like this" to indicate direct quotes from your manuscript), or elaborate on this item by providing additional information not in the ms, or briefly explain why the item is not applicable/relevant for your study

Not applicable; all analyses followed hypotheses clearly stated at the end of the Introduction.

18-i) Subgroup analysis of comparing only users

A subgroup analysis of comparing only users is not uncommon in ehealth trials, but if done, it must be stressed that this is a self-selected sample and no longer an unbiased sample from a randomized trial (see 16-iii).

	1	2	3	4	5	
subitem not at all important	○	○	○	○	◉	essential

Does your paper address subitem 18-i?

Copy and paste relevant sections from the manuscript (include quotes in quotation marks "like this" to indicate direct quotes from your manuscript), or elaborate on this item by providing additional information not in the ms, or briefly explain why the item is not applicable/relevant for your study

Results section:
"Modules completed as moderator (Hypothesis 5)

...The number of modules completed moderated the level of improvement in primary control from baseline to post-intervention for the intervention group (b = 1.420, 95% CIs: 0.422, 2.418, P = .01, Cohen's d = 0.389), such that primary control improved further with every additional module completed. Number of modules completed did not moderate any of the other studied variables (all remaining Ps > .05 and Cohen's d values < .24).

User feedback (Hypothesis 6)
The overall quality of the app was rated highly, with a mean score of 3.94 out of a maximum score of 5 (SD=0.58). Participants rated their subjective quality of the app slightly lower (M=3.19, SD=0.85). Within the subjective quality subscale, participants expressed that they would not choose to pay for the app (M=2.22, SD=1.14), which was the only item to be rated with a mean score below 2.5. The app was rated particularly positively for its functionality (M=4.19, SD=0.75), information (M=3.96, SD=0.63), and aesthetics (M=3.95, SD=0.63). Although all subscales were rated highly, the engagement subscale achieved the lowest mean score (M=3.68, SD=0.65). Within the engagement subscale, the items assessing customisation and interactivity were rated the lowest (M=3.31, SD=0.85; M=3.47, SD=0.82, respectively)."

19) All important harms or unintended effects in each group

(for specific guidance see CONSORT for harms)

Does your paper address CONSORT subitem 19? *

Copy and paste relevant sections from the manuscript (include quotes in quotation marks "like this" to indicate direct quotes from your manuscript), or elaborate on this item by providing additional information not in the ms, or briefly explain why the item is not applicable/relevant for your study

No harms were identified in this study.

19-i) Include privacy breaches, technical problems

Include privacy breaches, technical problems. This does not only include physical "harm" to participants, but also incidents such as perceived or real privacy breaches [1], technical problems, and other unexpected/unintended incidents. "Unintended effects" also includes unintended positive effects [2].

	1	2	3	4	5	
subitem not at all important	○	○	○	○	●	essential

Does your paper address subitem 19-i?

Copy and paste relevant sections from the manuscript (include quotes in quotation marks "like this" to indicate direct quotes from your manuscript), or elaborate on this item by providing additional information not in the ms, or briefly explain why the item is not applicable/relevant for your study

No privacy breaches or technical problems were identified during the study.

19-ii) Include qualitative feedback from participants or observations from staff/researchers

Include qualitative feedback from participants or observations from staff/researchers, if available, on strengths and shortcomings of the application, especially if they point to unintended/unexpected effects or uses. This includes (if available) reasons for why people did or did not use the application as intended by the developers.

	1	2	3	4	5	
subitem not at all important	○	○	○	●	○	essential

Does your paper address subitem 19-ii?

Copy and paste relevant sections from the manuscript (include quotes in quotation marks "like this" to indicate direct quotes from your manuscript), or elaborate on this item by providing additional information not in the ms, or briefly explain why the item is not applicable/relevant for your study

We included a measure of app quality and an associated hypothesis about positive experience of the app (Hypothesis 6). From the Results section:

"User feedback (Hypothesis 6)
The overall quality of the app was rated highly, with a mean score of 3.94 out of a maximum score of 5 (SD=0.58). Participants rated their subjective quality of the app slightly lower (M=3.19, SD=0.85). Within the subjective quality subscale, participants expressed that they would not choose to pay for the app (M=2.22, SD=1.14), which was the only item to be rated with a mean score below 2.5. The app was rated particularly positively for its functionality (M=4.19, SD=0.75), information (M=3.96, SD=0.63), and aesthetics (M=3.95, SD=0.63). Although all subscales were rated highly, the engagement subscale achieved the lowest mean score (M=3.68, SD=0.65). Within the engagement subscale, the items assessing customisation and interactivity were rated the lowest (M=3.31, SD=0.85; M=3.47, SD=0.82, respectively)."

DISCUSSION

22) Interpretation consistent with results, balancing benefits and harms, and considering other relevant evidence

NPT: In addition, take into account the choice of the comparator, lack of or partial blinding, and unequal expertise of care providers or centers in each group

22-i) Restate study questions and summarize the answers suggested by the data, starting with primary outcomes and process outcomes (use)

Restate study questions and summarize the answers suggested by the data, starting with primary outcomes and process outcomes (use).

	1	2	3	4	5	
subitem not at all important	○	○	○	○	◉	essential

Does your paper address subitem 22-i? *

Copy and paste relevant sections from the manuscript (include quotes in quotation marks "like this" to indicate direct quotes from your manuscript), or elaborate on this item by providing additional information not in the ms, or briefly explain why the item is not applicable/relevant for your study

"The purpose of this study was to evaluate the efficacy of a mobile app-based, self-directed psychological intervention for individuals providing care to family or friends with a physical or mental condition. The sample consisted predominantly of mothers of children with a disability with high levels of care burden and stress. The intervention group experienced improvements in the primary outcomes of stress, depression, anxiety, and subjective wellbeing across the intervention period despite using only a small number of the treatment modules offered, with further improvements in mental health and outlook observed over the three-to-four month follow-up period. Participants rated the intervention app highly for its usability and quality, with potential to improve the app design further through the addition of greater personalisation and flexibility. Given the limited number of studies that have investigated the potential of mHealth tools for caregiver populations, the current results have important implications for future work in this field."

22-ii) Highlight unanswered new questions, suggest future research

Highlight unanswered new questions, suggest future research.

	1	2	3	4	5	
subitem not at all important	○	○	○	○	◉	essential

Does your paper address subitem 22-ii?

Copy and paste relevant sections from the manuscript (include quotes in quotation marks "like this" to indicate direct quotes from your manuscript), or elaborate on this item by providing additional information not in the ms, or briefly explain why the item is not applicable/relevant for your study

Future research directions are discussed in light of observed findings:

"Further exploration of ways to reduce intervention-related workload whilst ensuring positive outcomes is needed. Testing of which modules are most efficacious may provide data for the StressLess to recommend specific combinations of modules as most important. Augmenting longer modules, as per StressLess, with micro-intervention content, may also help to provide immediate symptom relief when needed, but without unrealistic time commitments [e.g., 74].
...Future research could consider the utility of providing a tailored experience to app users, such that the program dynamically adapts to a participant's context and usage. Based on mood assessments within the app, and automatically detected usage patterns, the app could in future send push notifications with recommendations to engage in specific modules at a given point in time [e.g., 86,87]. Such tailoring, based on knowledge of a user's past behaviour, is common in consumer applications (e.g., Netflix) and may provide similar benefits to users of mHealth interventions by providing support at the time of need, based on previous usage behaviour [88,89]. Such systems would benefit from a participatory design to ensure that the intelligent health system adequately balances automated decision-making with the user's own input, and that its design is also in awareness of privacy concerns that participants may have in disclosing personal data. Developing intelligent and adaptive mHealth interventions through emerging big data technologies such as machine learning may be a promising avenue for future research with this population [88,90,91,92]. "

20) Trial limitations, addressing sources of potential bias, imprecision, and, if relevant, multiplicity of analyses

20-i) Typical limitations in ehealth trials

Typical limitations in ehealth trials: Participants in ehealth trials are rarely blinded. Ehealth trials often look at a multiplicity of outcomes, increasing risk for a Type I error. Discuss biases due to non-use of the intervention/usability issues, biases through informed consent procedures, unexpected events.

	1	2	3	4	5	
subitem not at all important	○	○	○	◉	○	essential

Does your paper address subitem 20-i? *

Copy and paste relevant sections from the manuscript (include quotes in quotation marks "like this" to indicate direct quotes from your manuscript), or elaborate on this item by providing additional information not in the ms, or briefly explain why the item is not applicable/relevant for your study

We note a range of limitations, many related to the app-based nature of the study:
"The current study has a number of limitations... Second, given the nature of the intervention, blinding to condition was not possible. This may have impacted results as participants could reasonably predict the researchers' hypotheses. Third, as the study was limited to individuals with an iOS-based phone, it is unclear whether usage patterns and user experiences will generalise to Android users. In Australia, marketshare is reasonably even for iOS and Android-based smartphones [93-94], [REF], but this is not the case globally. At the very least, this impedes uptake of the StressLess intervention. It may also signal different demographics that may relate to efficacy; an issue that needs further exploration in eHealth interventions. Fourth, although sample sizes were adequate as calculated through a priori power analyses, attrition across the study duration resulted in smaller numbers by the final assessment..."

21) Generalisability (external validity, applicability) of the trial findings

NPT: External validity of the trial findings according to the intervention, comparators, patients, and care providers or centers involved in the trial

21-i) Generalizability to other populations

Generalizability to other populations: In particular, discuss generalizability to a general Internet population, outside of a RCT setting, and general patient population, including applicability of the study results for other organizations

	1	2	3	4	5	
subitem not at all important	○	○	○	○	◉	essential

Does your paper address subitem 21-i?

Copy and paste relevant sections from the manuscript (include quotes in quotation marks "like this" to indicate direct quotes from your manuscript), or elaborate on this item by providing additional information not in the ms, or briefly explain why the item is not applicable/relevant for your study

This is our first limitation in the Discussion:
"The current study has a number of limitations. First, as noted above, the sample is not broadly representative of caregivers in national studies [2]. This sampling bias may reflect the recruitment and intervention delivery methods of the current study through technology, such as social media and smartphones. The results may therefore not generalise to other caregiver contexts, particularly to caregivers who face barriers in accessing technology. Future research could aim to examine the effectiveness of mHealth interventions within different caring contexts."

21-ii) Discuss if there were elements in the RCT that would be different in a routine application setting

Discuss if there were elements in the RCT that would be different in a routine application setting (e.g., prompts/reminders, more human involvement, training sessions or other co-interventions) and what impact the omission of these elements could have on use, adoption, or outcomes if the intervention is applied outside of a RCT setting.

	1	2	3	4	5	
subitem not at all important	○	○	○	◉	○	essential

Does your paper address subitem 21-ii?

Copy and paste relevant sections from the manuscript (include quotes in quotation marks "like this" to indicate direct quotes from your manuscript), or elaborate on this item by providing additional information not in the ms, or briefly explain why the item is not applicable/relevant for your study

In the Discussion, we emphasise the need to consider tailoring. At the end of this passage, we make it clear that doing so may better approximate the face-to-face therapy experience:

"Future research could consider the utility of providing a tailored experience to app users, such that the program dynamically adapts to a participant's context and usage. Based on mood assessments within the app, and automatically detected usage patterns, the app could in future send push notifications with recommendations to engage in specific modules at a given point in time [e.g., 86,87]. Such tailoring, based on knowledge of a user's past behaviour, is common in consumer applications (e.g., Netflix) and may provide similar benefits to users of mHealth interventions by providing support at the time of need, based on previous usage behaviour [88,89]. Such systems would benefit from a participatory design to ensure that the intelligent health system adequately balances automated decision-making with the user's own input, and that its design is also in awareness of privacy concerns that participants may have in disclosing personal data. Developing intelligent and adaptive mHealth interventions through emerging big data technologies such as machine learning may be a promising avenue for future research with this population [88,90,91,92]. This active prompting may also better approximate the structure and support provided in face-to-face therapy."

OTHER INFORMATION

23) Registration number and name of trial registry

Does your paper address CONSORT subitem 23? *

Copy and paste relevant sections from the manuscript (include quotes in quotation marks "like this" to indicate direct quotes from your manuscript), or elaborate on this item by providing additional information not in the ms, or briefly explain why the item is not applicable/relevant for your study

"This study was approved by the Deakin University Human Research Ethics Committee (2016-151), and registered with the Australian New Zealand Clinical Trials Registry (ACTRN12616000996460)."

24) Where the full trial protocol can be accessed, if available

Does your paper address CONSORT subitem 24? *

Cite a Multimedia Appendix, other reference, or copy and paste relevant sections from the manuscript (include quotes in quotation marks "like this" to indicate direct quotes from your manuscript), or elaborate on this item by providing additional information not in the ms, or briefly explain why the item is not applicable/relevant for your study

"This study was approved by the Deakin University Human Research Ethics Committee (2016-151), and registered with the Australian New Zealand Clinical Trials Registry (ACTRN12616000996460)."

25) Sources of funding and other support (such as supply of drugs), role of funders

Does your paper address CONSORT subitem 25? *

Copy and paste relevant sections from the manuscript (include quotes in quotation marks "like this" to indicate direct quotes from your manuscript), or elaborate on this item by providing additional information not in the ms, or briefly explain why the item is not applicable/relevant for your study

"Funding
The research was funded by the Deakin University – Australian Unity wellbeing partnership. "
The financial partner did not place constraints on what could be published.

X27) Conflicts of Interest (not a CONSORT item)

X27-i) State the relation of the study team towards the system being evaluated

In addition to the usual declaration of interests (financial or otherwise), also state the relation of the study team towards the system being evaluated, i.e., state if the authors/evaluators are distinct from or identical with the developers/sponsors of the intervention.

	1	2	3	4	5	
subitem not at all important	○	○	○	○	◉	essential

Does your paper address subitem X27-i?

Copy and paste relevant sections from the manuscript (include quotes in quotation marks "like this" to indicate direct quotes from your manuscript), or elaborate on this item by providing additional information not in the ms, or briefly explain why the item is not applicable/relevant for your study

In the Acknowledgements section, we outline that the project was made possible by industry funding. We also state that there are no conflicts of interest as we do not have a financial stake in the app (it is freely available to the public).

About the CONSORT EHEALTH checklist

As a result of using this checklist, did you make changes in your manuscript? *

- ○ yes, major changes
- ◉ yes, minor changes
- ○ no

What were the most important changes you made as a result of using this checklist?

Minor textual changes to enhance clarity about recruitment and key design features of the program

How much time did you spend on going through the checklist INCLUDING making changes in your manuscript *

60 minutes

As a result of using this checklist, do you think your manuscript has improved? *

- (selected) yes
- no
- Other:

Would you like to become involved in the CONSORT EHEALTH group?

This would involve for example becoming involved in participating in a workshop and writing an "Explanation and Elaboration" document

- yes
- (selected) no
- Other:

Any other comments or questions on CONSORT EHEALTH

nil

STOP - Save this form as PDF before you click submit

To generate a record that you filled in this form, we recommend to generate a PDF of this page (on a Mac, simply select "print" and then select "print as PDF") before you submit it.

When you submit your (revised) paper to JMIR, please upload the PDF as supplementary file.

Don't worry if some text in the textboxes is cut off, as we still have the complete information in our database. Thank you!

Final step: Click submit !

Click submit so we have your answers in our database!

Submit

Never submit passwords through Google Forms.

This content is neither created nor endorsed by Google. Report Abuse - Terms of Service - Privacy Policy

Google Forms
